# Extracellular Vesicle‐Packaged Linc‐ZNF25‐1 from Pancreatic Cancer Cell Promotes Pancreatic Stellate Cell Uptake of Asparagine to Advance Chemoresistance

**DOI:** 10.1002/advs.202413439

**Published:** 2025-03-05

**Authors:** Miao Yu, Mingxin Su, Zhenfeng Tian, Lele Pan, Zongmeng Li, Enlai Huang, Yinting Chen

**Affiliations:** ^1^ Guangdong Provincial Key Laboratory of Malignant Tumor Epigenetics and Gene Regulation Department of Gastroenterology Sun Yat‐Sen Memorial Hospital Sun Yat‐Sen University Guangzhou 510120 P. R. China

**Keywords:** asparagine, extracellular vesicles, long noncoding RNA, pancreatic cancer, pancreatic stellate cells

## Abstract

Extensive fibrous stroma plays an important role in gemcitabine (GEM) resistance. However, the mechanism by which pancreatic cancer cells interact with pancreatic stellate cells (PSCs) to promote GEM resistance remains unclear. This study investigates the role of metabolic crosstalk between these two cells in inducing GEM resistance. Extracellular vesicles (EVs) of parental and GEM‐resistant pancreatic cancer cells are extracted and performed metabolic assays and long noncoding RNA (lncRNA) sequencing. Pancreatic cancer cell‐derived EVs promote PSCs activation and extracellular matrix formation, and GEM‐resistant pancreatic cancer cells produce more asparagine (Asn), favoring PSCs activation. Mechanistically, pancreatic cancer cell‐derived EVs mediate linc‐ZNF25‐1 to promote Asn uptake via the IGF2BP3/c‐Myc/SLC1A5 pathway in PSCs. In addition, mouse models elucidate the oncogenic function of linc‐ZNF25‐1 and the enhanced therapeutic effect of asparaginase (L‐ASNase) in combination with GEM in pancreatic cancer. This study demonstrates that pancreatic cancer cell‐derived EVs promote the uptake of Asn released from pancreatic cancer cells through the upregulation of SLC1A5 in PSCs, facilitating PSCs activation and pancreatic cancer resistance to GEM. L‐ASNase in combination with GEM is a potential therapeutic strategy for targeting stromal cells to enhance the efficacy of chemotherapeutic agents against pancreatic cancer.

## Graphical abstract

This study explores the role of gemcitabine (GEM)‐resistant pancreatic cancer‐derived extracellular vesicles (EVs) in activating pancreatic stellate cells (PSCs) and promoting extracellular matrix production. GEM‐resistant cells release higher levels of asparagine (Asn), which triggers PSC activation. Long noncoding RNA sequencing identified linc‐ZNF25‐1 transfer via EVs, enhancing Asn uptake through the IGF2BP3/c‐Myc/SLC1A5 pathway. In vivo, asparaginase combined with GEM show enhanced therapeutic effects.

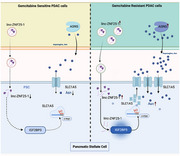

## 1 Introduction

Pancreatic cancer is a malignant tumor with a high mortality rate, and most tumors are diagnosed in advanced stages.^[^ ^1^ ^]^ Gemcitabine (GEM)‐based single‐agent or combination chemotherapy is an important therapeutic strategy for treating patients with advanced pancreatic cancer.^[^ ^2^ ^]^ However, the gradual development of GEM resistance severely limits the effectiveness of treatment.^[^ ^3^ ^]^ In addition to cancer cells, the abundant mesenchymal components in pancreatic cancer promote drug resistance. Cancer‐associated fibroblasts (CAF) and their deposited extracellular matrix are important for the formation of an extensive and dense fibrous stroma in pancreatic cancer.^[^ ^4^ ^]^ In pancreatic cancer, a subset of CAFs originate from the activation of pancreatic stellate cells (PSCs). Direct or indirect interactions between pancreatic cancer cells and PSCs may play important roles in GEM resistance.^[^ ^5^ ^]^ Therefore, exploring the communication crosstalk between pancreatic cancer cells and PSCs is necessary for the treatment of patients with pancreatic cancer, as well as for the reduction of drug resistance.

In response to rapid tumor growth, most pancreatic cancer cells develop metabolic adaptations, such as the Warburg effect and glutamine addiction, to obtain nutrients from the tumor microenvironment (TME).^[^ ^6^ ^]^ Asparagine (Asn) is a non‐essential amino acid produced in an ATP‐dependent manner using aspartic acid and glutamine as substrates in the presence of asparagine synthetase (ASNS).^[^ ^7^ ^]^ The clinical use of asparaginase (L‐ASNase) to deplete Asn and, thereby, treat leukemia suggests that Asn plays an important role in tumor cells.^[^ ^8^ ^]^ However, ASNase is not as effective against a variety of solid tumors, such as pancreatic cancer, possibly because of the presence of ASNS, which is consistently highly expressed in the pancreas,^[^ ^9^ ^]^ and metabolic signaling pathways that induce ASNS expression.^[^ ^10^ ^]^ Previous studies have shown that many tumor cells can produce and release Asn.^[^ ^11^ ^]^ However, its effect on PSCs, which occupy a large portion of the mesenchymal component in pancreatic cancer, is unclear. This underscores the necessity for further investigation into the influence of Asn on PSCs activation and the tumor microenvironment, given the critical role of PSCs in tumor progression and drug resistance.

Extracellular vesicles (EVs), including exosomes, play an important role in the exchange of materials and information between cells.^[^ ^12^ ^]^ There is growing evidence that EVs are packed with substances such as lipids, proteins, and nucleic acids, and can transmit biological signals from one cell type or tissue to another.^[^ ^13^ ^]^ They are effective signaling molecules between cancer cells and cells in the TME and play an important role in chemoresistance in various tumors.^[^ ^14^ ^]^ Notably, EVs derived from cholangiocarcinoma cells can facilitate the formation of tumor stroma by inducing the fibroblastic differentiation of mesenchymal stem cells (MSCs).^[^ ^15^ ^]^ Similarly, research revealed that melanoma cell‐derived EVs transfer miR‐92b‐3p to fibroblasts through the uptake of EV cargo, triggering the formation of CAFs by targeting PTEN.^[^ ^16^ ^]^ The activation of PSCs into CAFs is an important step in the development of fibrogenesis. CAFs are intrinsically insensitive to GEM and play a crucial role in the development of resistance to tumor therapy.^[^ ^17^ ^]^ Consequently, pancreatic cancer‐derived EVs that influence PSCs activation may constitute a key mechanism underlying drug resistance.

This study aims to elucidate the mechanisms underlying the interactions between pancreatic cancer cells and PSCs, particularly focusing on how EVs mediate the transfer of metabolites that contribute to GEM resistance. By investigating the differential metabolite Asn and its role in the activation of PSCs, we seek to provide insights into metabolic communication within the tumor microenvironment. Furthermore, we will explore the regulatory impact of long noncoding RNAs (lncRNAs) in this context, specifically how pancreatic cancer cells modulate the IGF2BP3/c‐Myc/SLC1A5 signaling pathway through EV‐mediated linc‐ZNF25‐1 to enhance Asn uptake by PSCs. Ultimately, this research aims to contribute to a deeper understanding of the mechanisms driving chemoresistance in pancreatic cancer and to identify potential therapeutic strategies for targeting the mesenchymal microenvironment, thereby improving treatment outcomes for patients.

## 2 Results

### 2.1 GEM‐Resistant Pancreatic Cancer Cell‐Derived EVs more Efficiently Promote PSCs Activation

**Figure 1:**
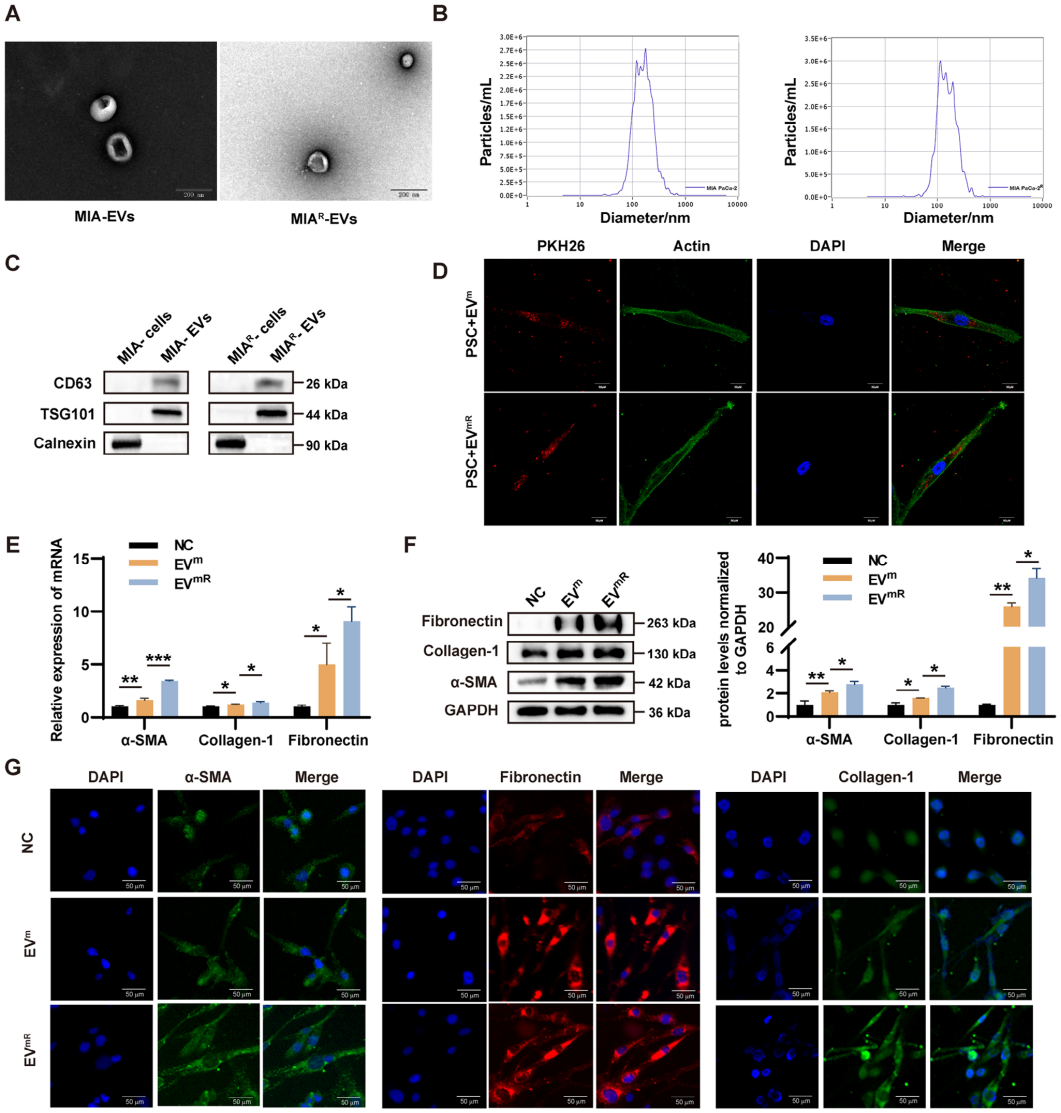
GEM‐resistant pancreatic cancer cell‐derived EVs more efficiently promote PSCs activation. A) Representative TEM images of EVs isolated from MIA PaCa‐2/MIA PaCa‐2^R^ supernatants. B) Nanoparticle tracking analysis (NTA) of the size distribution and number of EVs. C) Western blot showing the expression of the EVs markers CD63 and TSG101 and the negative protein marker Calnexin in the EVs and cell lysates. D) Representative fluorescence images of Actin‐labeled (green) PSCs incubated with PKH26‐labeled (red) EVs for 24 h. E‐G) mRNA and protein expression of α‐SMA, Collagen‐1, and Fibronectin during incubation of PSCs with MIA PaCa‐2/MIA PaCa‐2^R^‐derived EVs and their immunofluorescence images; ^*^ *p* < 0.05; ^**^ *p* < 0.01; ^***^ *p* < 0.001.

To investigate the impact of pancreatic cancer cell‐derived EVs on PSCs, we first isolated human primary PSCs and established immortalized PSCs by co‐transfecting with the SV40 LT and hTERT immortalization viruses (Figure S1, Supporting Information). EVs were then isolated from MIA PaCa‐2/MIA PaCa‐2^R^ and PANC‐1/PANC‐1^R^ cells and characterized using transmission electron microscopy (TEM) and particle size analysis (**Figure** **1**A,B; Figure S2A,B, Supporting Information). The protein markers CD63 and TSG101 were highly expressed in EVs and extremely weak expression levels were detected in the corresponding cell lysates, in contrast to the EV‐negative protein marker calnexin (Figure 1C; Figure S2C, Supporting Information), further indicating the successful extraction of EVs. To confirm the uptake of EVs by PSCs, we applied PKH26 labeled EVs to immortalized PSCs, and the results showed that PSCs successfully internalized MIA PaCa‐2/MIA PaCa‐2^R^ derived EVs (Figure 1D). The growth of PSCs treated with different concentrations of EVs ranging from 0 to 100 µg ml^−1^ was measured using CCK‐8 to determine the optimal concentration of EVs for further experiments at 50 µg ml^−1^ (Figure S2D, Supporting Information).

To assess the activating effect of pancreatic cancer cell‐derived EVs on PSCs, we applied 50 µg ml^−1^ of MIA PaCa‐2/MIA PaCa‐2^R^ and PANC‐1/PANC‐1^R^‐derived EVs to PSCs. qRT‐PCR and western blot results showed that GEM‐resistant pancreatic cancer cell‐derived EVs promoted a significant upregulation of α‐SMA as well as an increase in Collagen‐1 and Fibronectin compared with parental cell‐derived EVs; immunofluorescence showed the same effect (Figure 1E–G; Figure S2E–G, Supporting Information). We previously established orthotopic tumor models in mice using MIA PaCa‐2 and MIA PaCa‐2^R^ cells.^[^ ^18^ ^]^ To further investigate the impact of GEM‐resistant pancreatic cancer cells on the activation of PSCs, we performed immunohistochemical staining on collected orthotopic tumors. The results showed more positive staining for α‐SMA as well as extracellular matrix proteins in GEM‐resistant tumors (Figure S2H, Supporting Information).

### 2.2 Enhanced Activation of PSCs by SLC1A5 Uptake of GEM‐Resistant Pancreatic Cancer Cell‐Derived Asn

**Figure 2:**
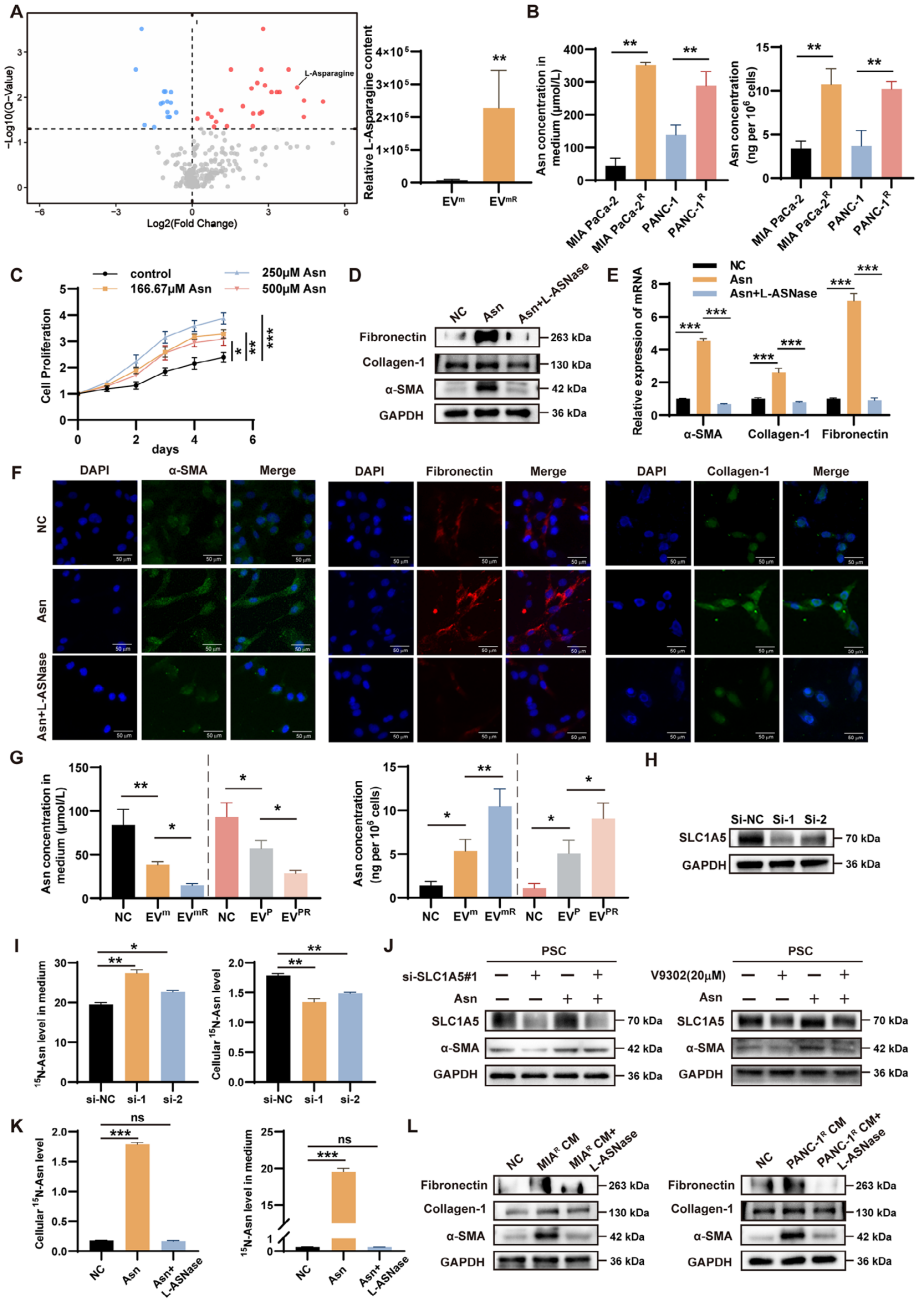
Enhanced activation of PSCs by SLC1A5 uptake of GEM‐resistant pancreatic cancer cell‐derived Asn. A) Volcano plots of metabolomics sequencing of MIA PaCa‐2/MIA PaCa‐2^R^‐derived EVs and the relative content of Asn. B) Concentrations of Asn in parental and GEM‐resistant pancreatic cancer cells and conditioned medium. C) Effects of different concentrations of Asn on the growth of PSCs. D‐F) qRT‐PCR, western blot, and immunofluorescence were performed to detect the effect of Asn with or without L‐ASNase (10 µg ml^−1^) on α‐SMA, Collagen‐1, and Fibronectin in PSCs. G) Medium and intracellular Asn content in PSCs treated with different EVs after the addition of Asn (250 µm) for 24 h. H) Western blot to verify the role of SLC1A5 small interfering RNAs (siRNAs). I) Effect of knockdown of SLC1A5 on the uptake of Asn by PSCs. J) Asn‐induced α‐SMA enhanced in PSCs can be reversed by si‐SLCA5 and V9302. K) Effect of L‐ASNase on the uptake of 15N‐Asn by PSCs. L) Effect on PSCs activation when cultured in GEM‐resistant pancreatic cancer cell‐conditioned medium with or without L‐ASNase (10 µg ml^−1^); ^*^ *p* < 0.05; ^**^ *p* < 0.01; ^***^ *p* < 0.001; ns: not significant.

To determine the possible metabolic associations of GEM‐resistant pancreatic cancer cell‐derived EVs that promote PSCs activation, we collected equal amounts of EV^m^ and EV^mR^ and performed metabolomic sequencing, which revealed that Asn was the most significantly different amino acid (**Figure** **2**A and **Table** **1**). Because ASNS is an important enzyme for the synthesis of Asn, qRT‐PCR was performed to determine the expression of ASNS in MIA PaCa‐2/MIA PaCa‐2^R^ and PANC‐1/PANC‐1^R^, expression was found to be significantly higher in GEM‐resistant cells (Figure S3A, Supporting Information). For further validation, concentrations of Asn in cells as well as in their respective conditioned media from MIA PaCa‐2/MIA PaCa‐2^R^ and PANC‐1/PANC‐1^R^ were measured, which showed that GEM‐resistant cells and conditioned media contained more Asn (Figure 2B). By determining the effect of different concentrations of Asn on the growth of PSCs, we found that 250 µm Asn significantly promoted the proliferation of PSCs (Figure 2C). To assess whether Asn promoted the activation of PSCs, we applied 250 µm Asn with or without L‐ASNase to PSCs. qRT‐PCR, western blot, and immunofluorescence demonstrated that Asn promoted the up‐regulation of α‐SMA, Collagen‐1, and Fibronectin, which could be inhibited by the addition of L‐ASNase (Figure 2D–F; Figure S3B, Supporting Information). PSC activation plays a crucial role in the progression of pancreatic cancer, with several signaling pathways, including MAPK, PI3K/AKT, and NF‐κB, shown to be central to this process.^[^ ^19^ ^]^ In this study, we found that Asn can promote PSCs activation. Based on this finding, we further explored the signaling pathways through which Asn induces PSC activation. Western blot analysis revealed that Asn activates the MAPK and AKT pathways, thereby promoting PSC activation (Figure S3C, Supporting Information).

**Table 1:** Top 5 upregulated or downregulated metabolites in EV^m^ and EV^mR^ Metabolomic Profiling.

Class	Metabolite	Change	Log2 [Fold Change]	Log10 [Q‐value]
Other	Phenol sulphate	up	5.5292	3.5155
Exogenous	Tetradecaethylene glycol(PEG‐14)	up	5.1496	2.616
Peptide	N,N,N‐Trimethyl‐alanyl‐proline betaine	up	4.5552	2.219
Lysoglycerophosphatidylcholine	LysoPC(0:0/16:0)	up	4.393	2.1148
Amino acid	L‐Asparagine	up	4.1324	2.1148
Amino acid	L‐Arginine	down	−2.2164	3.5155
Carbohydrate	Hexose(Glucose etc)	down	−1.9886	2.616
Amino acid	Methionine sulfoxide	down	−1.4933	2.1278
Acylcarnitine	Succinylcarnitine	down	−1.32	2.1278
Amino acid	L‐Methionine	down	−1.2306	1.8817

SLC1A5 mediates the cellular uptake of Asn;^[^ ^20^ ^]^ therefore, we determined the effect of pancreatic cancer cell‐derived EVs on SLC1A5 expression. Pancreatic cancer cell‐derived EVs upregulated SLC1A5 expression in PSCs, with a more pronounced effect in GEM‐resistant cells that was not altered by the addition of Asn alone (Figure S3D–E, Supporting Information). We also found that after the addition of a medium containing the same Asn content (250 µm) to EVs‐treated PSCs for 24 h, the GEM‐resistant cells‐EVs group had significantly lower Asn in the medium compared to the control group, while the intracellular content was significantly increased (Figure 2G). Consistent with this, the knockdown of SLC1A5 decreased the cellular uptake of Asn (Figure 2H–I; Figure S3F, Supporting Information). We also used V9302, a targeted inhibitor of SLC1A5, to inhibit SLC1A5 (Figure S3G, Supporting Information), and similarly, the Asn‐induced increase in α‐SMA, an indicator of PSCs activation, could be reversed by si‐SLCA5 and V9302 (Figure 2J; Figure S3H, Supporting Information).

The addition of L‐ASNase largely eliminated Asn from the cells and culture medium (Figure 2K). Notably, PSCs cultured in the GEM‐resistant pancreatic cancer cell‐conditioned medium showed enhanced activation (Figure S3I, Supporting Information), whereas L‐ASNase supplementation completely reversed this effect, demonstrating the involvement of pancreatic cancer cell‐derived Asn in this process (Figure 2L; Figure S3J, Supporting Information). To further illustrate that SLC1A5 correlates with the uptake of Asn by PSCs to promote activation, we treated pancreatic cancer mice with PBS or V9302, respectively, and immunohistochemical staining of their tumors showed that both SLC1A5 and α‐SMA were attenuated in the V9302‐treated group (Figure S3K, Supporting Information). In addition, immunofluorescence of previously obtained GEM‐resistant tumors showed increased expression of both α‐SMA and SLC1A5, though these proteins were primarily localized in different cellular regions (Figure S3L, Supporting Information).

### 2.3 Upregulation of SLC1A5 in PSCs by Linc‐ZNF25‐1 Mediated by Pancreatic Cancer Cell‐Derived EVs

**Figure 3:**
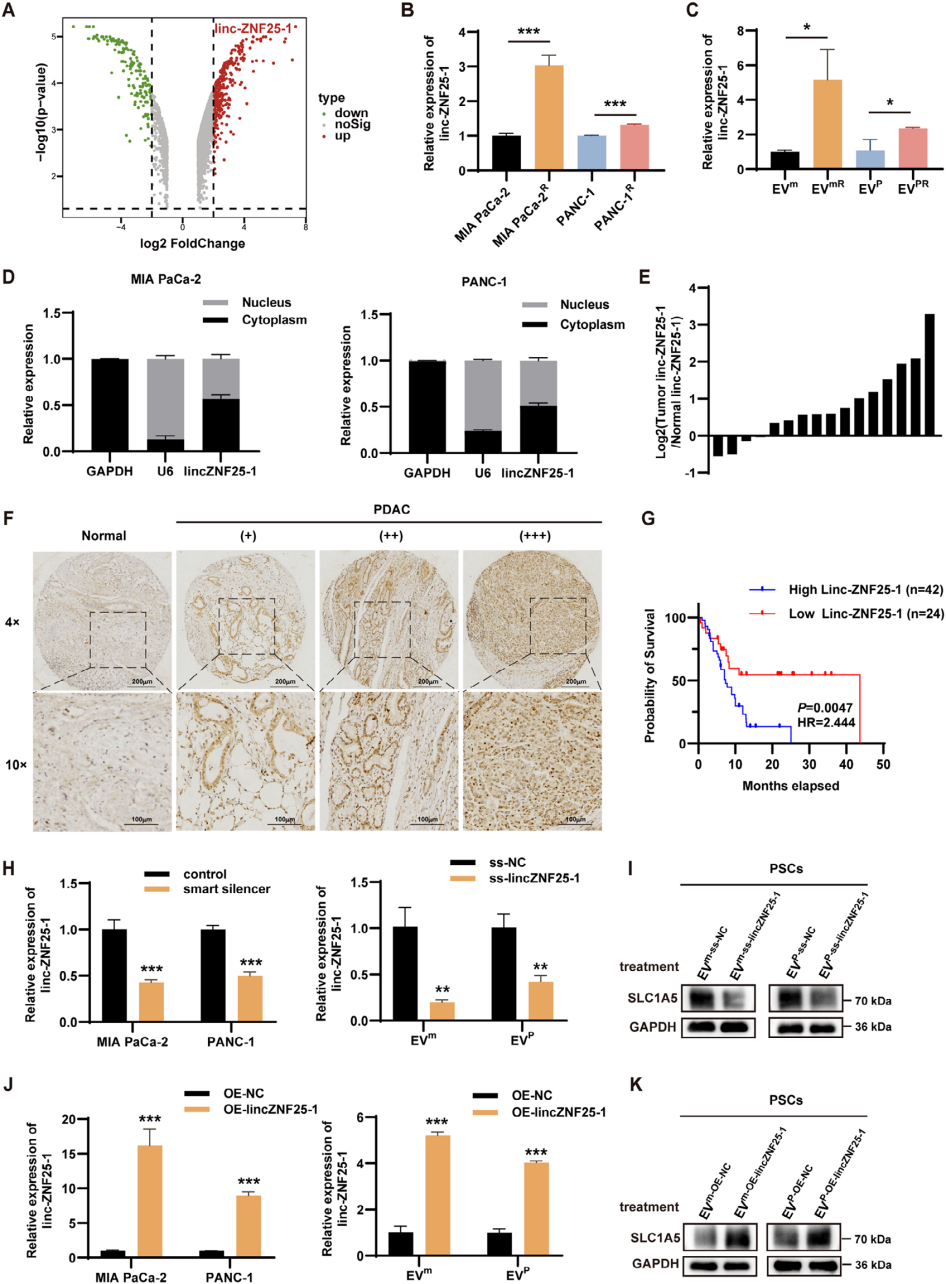
Upregulation of SLC1A5 in PSCs by linc‐ZNF25‐1 mediated by pancreatic cancer cell‐derived EVs. A) Volcano plot of lncRNA sequencing of MIA PaCa‐2/MIA PaCa‐2^R^‐derived EVs. B‐C) qRT‐PCR detection of linc‐ZNF25‐1 expression in MIA PaCa‐2/MIA PaCa‐2^R^ and PANC‐1/PANC‐1^R^ and their corresponding EVs. D) linc‐ZNF25‐1 expression in the nucleus and cytoplasm of pancreatic cancer cells. E) qRT‐PCR analysis of linc‐ZNF25‐1 expression in tumor and adjacent normal tissues from pancreatic cancer patients. F) RNA in situ hybridization of linc‐ZNF25‐1 in pancreatic cancer tissues (n = 66). G) Survival analysis of pancreatic cancer patients based on linc‐ZNF25‐1 expression. H) qRT‐PCR to detect the role of smart silencer silencing of linc‐ZNF25‐1 and the level of linc‐ZNF25‐1 in the corresponding EVs. I) Western blot analysis demonstrating that pancreatic cancer cell‐derived EVs with linc‐ZNF25‐1 knockdown reduced the upregulation of SLC1A5 compared to controls. J) qRT‐PCR to detect the efficiency of plasmid overexpression of linc‐ZNF25‐1 and the corresponding EVs in the levels of linc‐ZNF25‐1. K) Western blot analysis showing that EVs with linc‐ZNF25‐1 overexpression significantly increased SLC1A5 levels; ^*^ *p* < 0.05; ^**^ *p* < 0.01; ^***^ *p* < 0.001.

We performed lncRNA sequencing of MIA PaCa‐2‐and MIA PaCa‐2^R^‐derived EVs to discover the possible mechanisms by which EVs promote SLC1A5 upregulation in PSCs. The analysis identified linc‐ZNF25‐1 as the most significantly upregulated gene (**Figure** **3**A and **Table** **2**). Furthermore, we verified the differential expression of linc‐ZNF25‐1 in MIA PaCa‐2/MIA PaCa‐2^R^ and PANC‐1/PANC‐1^R^ by qRT‐PCR, and the results showed that there was more linc‐ZNF25‐1 in both GEM‐resistant cells and EVs derived from it (Figure 3B,C). Increased linc‐ZNF25‐1 expression was observed after treatment of PSCs with parental and GEM‐resistant pancreatic cancer cell‐derived EVs (Figure S4A, Supporting Information). Nucleoplasmic segregation showed that linc‐ZNF25‐1 was distributed in both the nucleus and cytoplasm, with more localization in the cytoplasm, which was consistent with its transport via EVs (Figure 3D). Further analysis using qRT‐PCR revealed that linc‐ZNF25‐1 was significantly upregulated in tumor tissues compared to adjacent normal tissues in 16 pancreatic cancer patients (Figure 3E). RNA in situ hybridization on a tissue microarray of 66 pancreatic cancer samples confirmed high linc‐ZNF25‐1 expression in tumors (Figure 3F). Clinical correlation showed that high linc‐ZNF25‐1 expression was associated with advanced tumor N stage and elevated CA199 levels (**Table** **3**). Survival analysis showed that high linc‐ZNF25‐1 expression was linked to poor prognosis (Figure 3G). Additionally, to account for the potential confounding effects of both N stage and CA199, we adjusted for these factors in the Cox regression analysis. The results demonstrated that linc‐ZNF25‐1 expression is an independent prognostic factor for survival (Figure S4B, Supporting Information).

**Table 2:** Top 5 upregulated or downregulated RNAs in EV^m^ and EV^mR^ lncRNA sequencing.

Gene	Change	Log2[Fold Change]	*P*.adj
linc‐ZNF25‐1	up	7.3347623	6.11E‐06
linc‐FCGR1B‐7	up	7.12099485	3.17E‐05
linc‐PARD6G‐2	up	6.6839292	1.11E‐05
linc‐POTED‐9	up	6.61700775	1.30E‐05
linc‐RAB23‐2	up	5.9001087	3.74E‐05
n335614	down	−7.0953915	6.11E‐06
linc‐IRAK4‐2	down	−6.2410346	6.12E‐06
n335636	down	−6.1326828	1.11E‐05
linc‐RGS2	down	−6.079342	1.11E‐05
n343017	down	−6.05586495	6.12E‐06

**Table 3:** Correlation of linc‐ZNF25‐1 expression with clinical pathological features.

Basic Data	High Linc‐ZNF25‐1 [n=42]	Low Linc‐ZNF25‐1 [n=24]	*P*
**Sex**			0.4183
Male	27	13	
Female	15	11	
**Age (years)**	61.95 ± 10.21	65.38 ± 7.988	0.1626
**Tobacco history**			0.7814
Yes	10	5	
No	32	19	
**Alcohol history**			0.7358
Yes	7	3	
No	35	21	
**Tumor location**			0.8417
Head and neck	29	16	
Body and tail	13	8	
**Grade**			0.9338
Low	9	6	
Middle	25	14	
High	8	4	
**Stage**			
**T**			0.2107
T1	2	1	
T2	8	8	
T3	25	8	
T4	7	7	
**N**			0.0051^*^
N0	16	18	
N1	26	6	
**M**			0.7547
M0	33	20	
M1	9	4	
**CA199**	312.5 (64.00,848.90)	129.10 (20.98,312.40)	0.0490^*^
**CA125**	20.35 (11.03,48.18)	15.90 (9.90,28.18)	0.3466
**CEA**	3.300 (2.125,4.900)	3.850 (2.075,4.925)	0.7229
**Ca^2+^**	2.310 (2.200,2.403)	2.255 (2.183,2.355)	0.2491
**CHO**	4.725 ± 1.797	4.42 ± 1.361	0.5241
**ALP**	150.00 (81.00,322.00)	100.50 (79.00,266.50)	0.4641
**Chemotherapy**			
Yes	22	10	0.4508
No	20	14	

^*^ *p* < 0.05;

CA199: carbohydrate antigen 19‐9, U/ml;

CA125: carbohydrate antigen 125, U/ml;

CEA: carcinoembryonic antigen, ng/ml;

CHO: serum total cholesterol, mmol/L;

ALP: alkaline phosphatase, U/L.

The efficiency of smart silencers in knocking down linc‐ZNF25‐1 in pancreatic cancer cells and the levels of linc‐ZNF25‐1 in the corresponding EVs were examined using qRT‐PCR (Figure 3H). PSCs treated with EVs from knockdown cells showed reduced linc‐ZNF25‐1 levels compared to controls (Figure S4C, Supporting Information). Meanwhile, we overexpressed linc‐ZNF25‐1 in pancreatic cancer cells by plasmid transfection, and the upregulation of linc‐ZNF25‐1 was detected at both the cellular and EVs levels (Figure 3J), an increase in linc‐ZNF25‐1 in PSCs was detected after the EVs from the overexpression group acted on PSCs (Figure S4D, Supporting Information).

To further explore whether EV‐mediated linc‐ZNF25‐1 affects SLC1A5 expression in PSCs, we extracted EVs from pancreatic cancer cells with knocked down or overexpressed linc‐ZNF25‐1 and applied them to PSCs. Western blotting demonstrated that EVs derived from linc‐ZNF25‐1 knockdown cells attenuated the upregulation of SLC1A5 compared to controls (Figure 3I), whereas EVs from linc‐ZNF25‐1 overexpressing cells significantly upregulated SLC1A5 (Figure 3K). Consistent with this, variations in the Asn uptake capacity of PSCs treated with different EVs correlated with the silencing or overexpression of linc‐ZNF25‐1 (Figure S4E,F, Supporting Information). To investigate whether linc‐ZNF25‐1 affects the synthesis and release of Asn, qRT‐PCR was performed to assess ASNS expression following linc‐ZNF25‐1 overexpression (Figure S4G, Supporting Information). The results showed that linc‐ZNF25‐1 overexpression does not affect ASNS expression. Furthermore, the Asn levels in both pancreatic cancer cells and their supernatants did not show significant differences (Figure S4H, Supporting Information).

### 2.4 Linc‐ZNF25‐1 Binds IGF2BP3 and Promotes IGF2BP3 Nuclear Translocation

**Figure 4:**
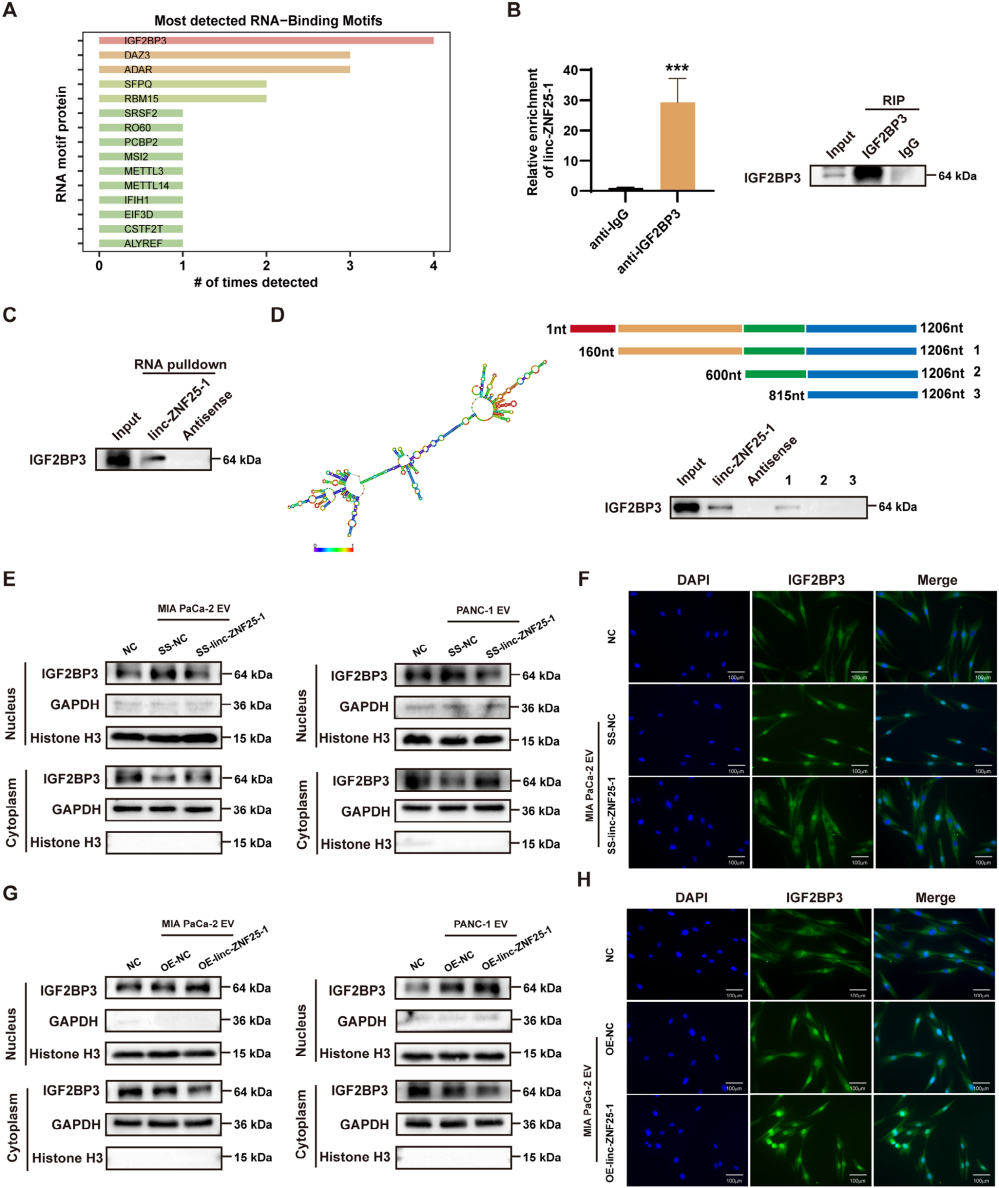
linc‐ZNF25‐1 binds IGF2BP3 and promotes IGF2BP3 nuclear translocation. A) Bioinformatics prediction of the possible binding proteins of linc‐ZNF25‐1. B) RIP assessment of the interaction between IGF2BP3 and linc‐ZNF25‐1 in PSCs cells treated with pancreatic cancer cell‐EVs. C) RNA pull‐down analysis of in vitro synthesized linc‐ZNF25‐1 probe interacting with IGF2BP3. D) RNA pull‐down assays were performed in PSCs to detect the interaction of a series of truncated sequences of linc‐ZNF25‐1 with IGF2BP3. E, G) Western blot analysis of IGF2BP3 after nucleoplasmic separation of PSCs cells treated with different EVs. F, H) Immunofluorescence staining shows changes in cytoplasmic and cytosolic localization of IGF2BP3 in PSCs cells treated with different EVs derived from MIA PaCa‐2; ^*^ *p* < 0.05; ^**^ *p* < 0.01; ^***^ *p* < 0.001.

Bioinformatic analysis predicted the possible binding proteins of linc‐ZNF25‐1, and the results showed that IGF2BP3 was the most promising binding protein (**Figure** **4**A). For further validation, an RNA immunoprecipitation (RIP) assay using an anti‐IGF2BP3 antibody was performed on PSCs treated with EVs from pancreatic cancer cells. RT‐qPCR confirmed the interaction between IGF2BP3 and linc‐ZNF25‐1 (Figure 4B; Figure S5A, Supporting Information). RNA pull‐down assay was performed to assess whether biotin‐labeled linc‐ZNF25‐1 could pull down the IGF2BP3 protein. Western blotting showed that, unlike biotin‐labeled antisense RNA, biotin‐labeled linc‐ZNF25‐1 pulled down the IGF2BP3 protein (Figure 4C). To identify the possible regions where linc‐ZNF25‐1 binds IFGF2BP3, we used bioinformatics analysis based on the minimum free energy (MFE) and segmentation function to establish the secondary structure of linc‐ZNF25‐1 and constructed a series of truncated versions of linc‐ZNF25‐1 accordingly. The RNA pull‐down results suggested that the 160–600 nt is critical in the interaction with IGF2BP3 (Figure 4D).

To investigate the alterations that occur upon linc‐ZNF25‐1 binding to IGF2BP3, we performed nucleoplasmic separation in different EVs‐treated PSCs and western blotting. An increase in IGF2BP3 nuclear translocation was observed in PSCs treated with pancreatic cancer cell‐EVs, whereas knockdown of linc‐ZNF25‐1 in pancreatic cancer cells did not promote IGF2BP3 nuclear translocation; in contrast, EVs overexpressing linc‐ZNF25‐1 in pancreatic cancer cells promoted an increase in IGF2BP3 nuclear translocation in PSCs (Figure 4E,G; Figure S5B–E, Supporting Information). Immunofluorescence confirmed that EVs containing linc‐ZNF25‐1 promoted IGF2BP3 nuclear translocation in PSCs (Figure 4F,H; Figure S5F,G, Supporting Information).

### 2.5 IGF2BP3 Up‐Regulates SLC1A5 by Stabilizing c‐Myc mRNA

**Figure 5:**
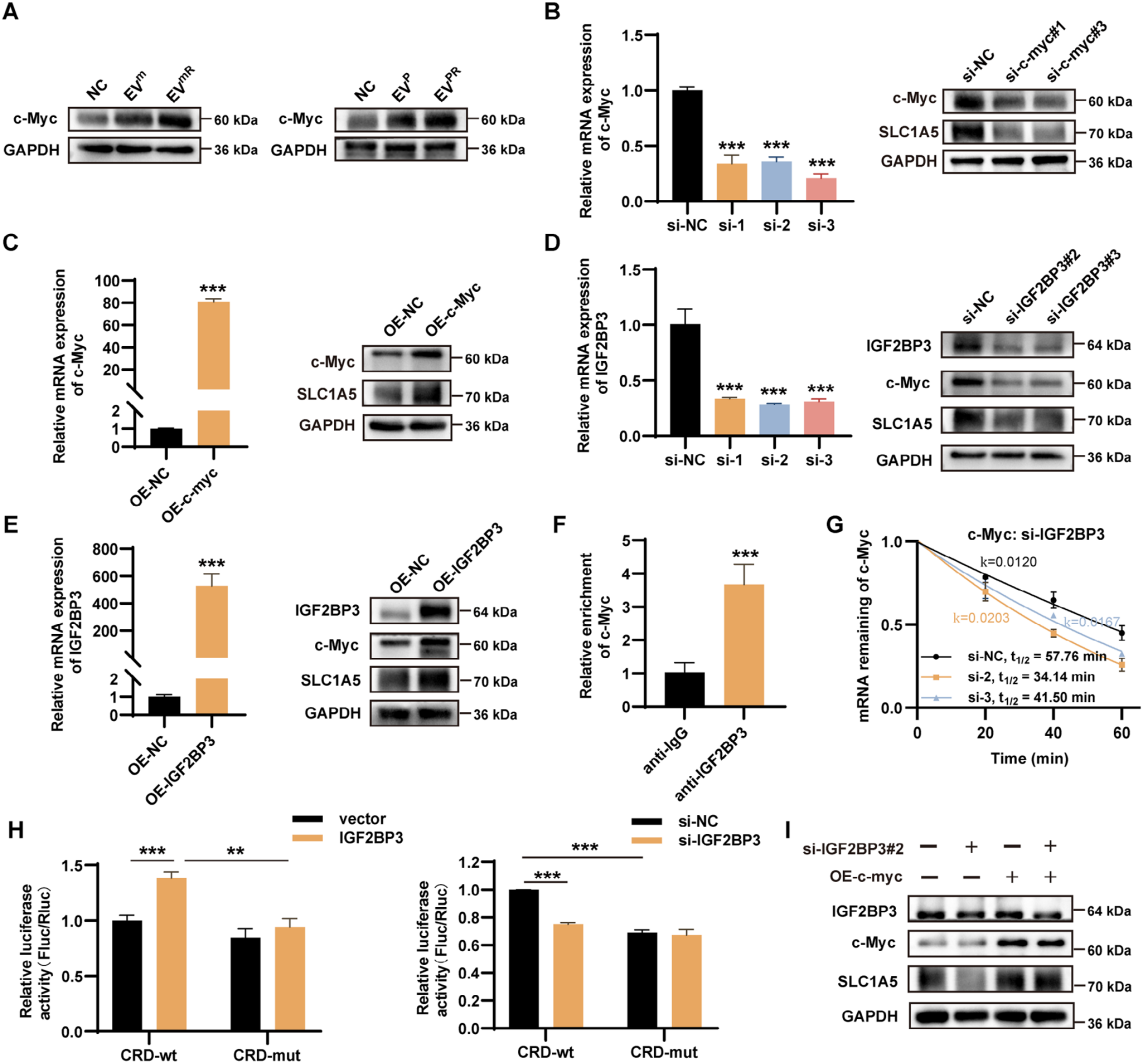
IGF2BP3 up‐regulates SLC1A5 by stabilizing c‐Myc mRNA. A) Protein expression of c‐Myc when PSCs were treated with EVs from parental or GEM‐resistant pancreatic cancer cells. B) Validation of small interfering RNA for c‐Myc and protein expression of SLC1A5 in PSCs when c‐Myc was knocked down. C) Validation of overexpression plasmid for c‐Myc and protein expression of SLC1A5 in PSCs when overexpressing c‐Myc. D) Validation of the silencing efficiency of IGF2BP3 and protein expression of c‐Myc and SLC1A5 in PSCs when knocking down IGF2BP3. E) The verification of the overexpression plasmid for IGF2BP3 and protein expression of c‐Myc and SLC1A5 in PSCs when overexpressing IGF2BP3. F) RIP‐qPCR indicated the interaction between IGF2BP3 and c‐Myc in PSCs cells. G) Knockdown of IGF2BP3 reduces c‐Myc RNA stability. H) Relative firefly luciferase (Fluc) activity of CRD‐wt or CRD‐mut reporters in PSCs ectopically expressing IGF2BP3 (left). Relative Fluc activity of CRD‐wt or CRD‐mut in PSCs with or without knockdown of IGF2BP3 (right). I) Protein expression of SLC1A5 was assayed in PSCs with or without knockdown of IGF2BP3 and with or without overexpression of c‐Myc; 𝑘: decay constant; ^*^ *p* < 0.05; ^**^ *p* < 0.01; ^***^ *p* < 0.001.

m6A (N6‐methyladenosine) is a key mRNA modification that regulates gene expression. IGF2BP3, an m6A reader, binds to m6A‐modified transcripts, enhancing their stability, with c‐Myc being not only a classical target of IGF2BP3 but also a regulatory protein for various transporter proteins.^[^ ^21^ ^]^ When pancreatic cancer cell‐derived EVs acted on PSCs, c‐Myc protein expression was upregulated, and this effect was more significant in GEM‐resistant pancreatic cancer cell‐derived EVs (**Figure** **5**A; Figure S6A, Supporting Information). After silencing the expression of c‐Myc with a small interfering RNA (siRNA), western blotting showed downregulation of SLC1A5 expression (Figure 5B; Figure S6C, Supporting Information). Similarly, SLC1A5 expression was upregulated when c‐Myc was overexpressed (Figure 5C; Figure S6B, Supporting Information). We silenced IGF2BP3 in PSCs and observed that the protein expression levels of c‐Myc and SLC1A5 were downregulated (Figure 5D; Figure S6E, Supporting Information). Similarly, IGF2BP3 overexpression upregulated the expression of c‐Myc and SLC1A5 (Figure 5E; Figure S6D, Supporting Information).

We performed RIP assays on PSCs cells using an anti‐IGF2BP3 antibody, and RIP‐qRT‐PCR showed an interaction between IGF2BP3 and c‐Myc (Figure 5F). Silencing of IGF2BP3 shortened the half‐life of c‐Myc mRNA (Figure 5G). The coding region instability determinant (CRD) in c‐Myc is essential for IGF2BP3 binding and is a region with high m6A abundance.^[^ ^21a^ ^]^ To further validate the binding of IGF2BP3 to c‐Myc, we inserted wild‐type or mutant CRD sequences into a dual‐luciferase reporter (Figure S6F, Supporting Information). Dual‐luciferase reporter gene assays showed that the relative Fluc activity of CRD‐wt, but not CRD‐mut, was increased by IGF2BP3 overexpression, whereas the relative Fluc activity of CRD‐wt was decreased in PSCs with IGF2BP3 knockdown (Figure 5H). Furthermore, the reduction in SLC1A5 induced by silencing IGF2BP3 was reversed by c‐Myc overexpression (Figure 5I; Figure S6G, Supporting Information).

### 2.6 Linc‐ZNF25‐1 Promotes Pancreatic Cancer Cell Proliferation and Drug Resistance In Vitro and In Vivo

**Figure 6:**
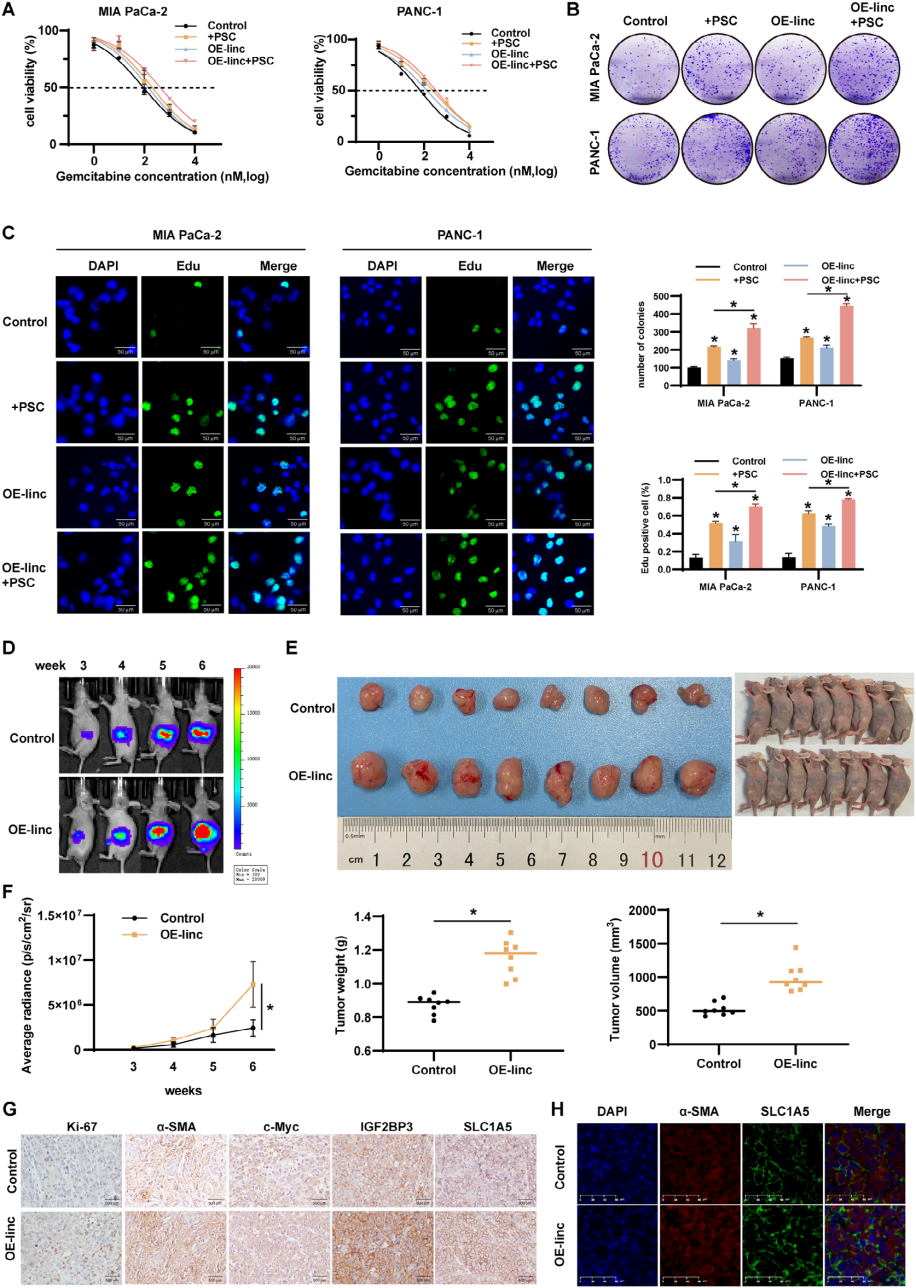
linc‐ZNF25‐1 promotes pancreatic cancer cell proliferation and drug resistance in vitro and in vivo. A) Cell viability of pancreatic cancer cells transfected or not with plasmid overexpressing linc‐ZNF25‐1 alone or co‐cultured with PSCs and then treated with different concentrations of gemcitabine for 72 h. B) Clone formation ability of pancreatic cancer cells after different treatments. C) EdU immunofluorescence demonstrates the proliferation of pancreatic cancer cells after different treatments. D) Representative bioluminescence images of in situ pancreatic cancer mice constructed with MIA PaCa‐2 cells transfected with negative control or overexpressing linc‐ZNF25‐1 lentivirus at a given time. E) Images of mice and tumors at the endpoint of the experiment. F) Mean radiation fluctuation curves over time and mouse tumor volume and weight. G) Immunohistochemistry of Ki‐67, α‐SMA, c‐Myc, IGF2BP3, and SLC1A5 of pancreatic cancer tumors in two groups of mice. H) Immunofluorescence co‐staining was performed for α‐SMA and SLC1A5 of tumors; ^*^ *p* < 0.05; ^**^ *p* < 0.01; ^***^ *p* < 0.001.

As linc‐ZNF25‐1, mediated by pancreatic cancer cell‐derived EVs, promotes PSC activation, the effect of linc‐ZNF25‐1 on pancreatic cancer cells was also evaluated. We co‐cultured pancreatic cancer cells transfected with or without the plasmid overexpressing linc‐ZNF25‐1 alone or with PSCs, followed by the addition of GEM at different concentrations. After 72 h, the CCK‐8 results showed that the overexpression of linc‐ZNF25‐1 promoted drug resistance in pancreatic cancer cells and was even more resistant after co‐culture with PSCs (**Figure** **6**A; Figure S7A,B, Supporting Information). Clone formation assays showed that overexpression of linc‐ZNF25‐1 or co‐culture with PSCs increased the proliferation of pancreatic cancer cells, and the combination of the two had a more significant effect (Figure 6B). Immunofluorescence after EdU incubation of pancreatic cancer cells with the above treatments showed similar results (Figure 6C).

We introduced the LV17 vector or LV17 – linc‐ZNF25‐1 lentivirus into MIA PaCa‐2 cells and injected them into the pancreas of nude mice to construct an in situ transplantation tumor model. Live imaging of small animals was performed at weeks 3, 4, 5, and 6 to show tumor growth, and bioluminescence images showed faster tumor growth in the LV17‐ linc‐ZNF25‐1 group (Figure 6D). Tumors were obtained after reaching the experimental endpoint, and the average radiation, tumor volume, and tumor weight of the LV17‐ linc‐ZNF25‐1 group were significantly higher than those of the control group (Figure 6E,F). Immunohistochemistry for Ki‐67, α‐SMA, c‐Myc, IGF2BP3, and SLC1A5 was performed on the obtained tumors, which showed more positive staining in the LV17‐ linc‐ZNF25‐1 group (Figure 6G). α‐SMA and SLC1A5 were co‐stained for immunofluorescence to assess the promoting effect of linc‐ZNF25‐1 on SLC1A5 in PSCs (Figure 6H).

### 2.7 Validation of GEM Combined with L‐ASNase to Enhance the Therapeutic Effect of Pancreatic Cancer In Vivo and In Vitro

**Figure 7:**
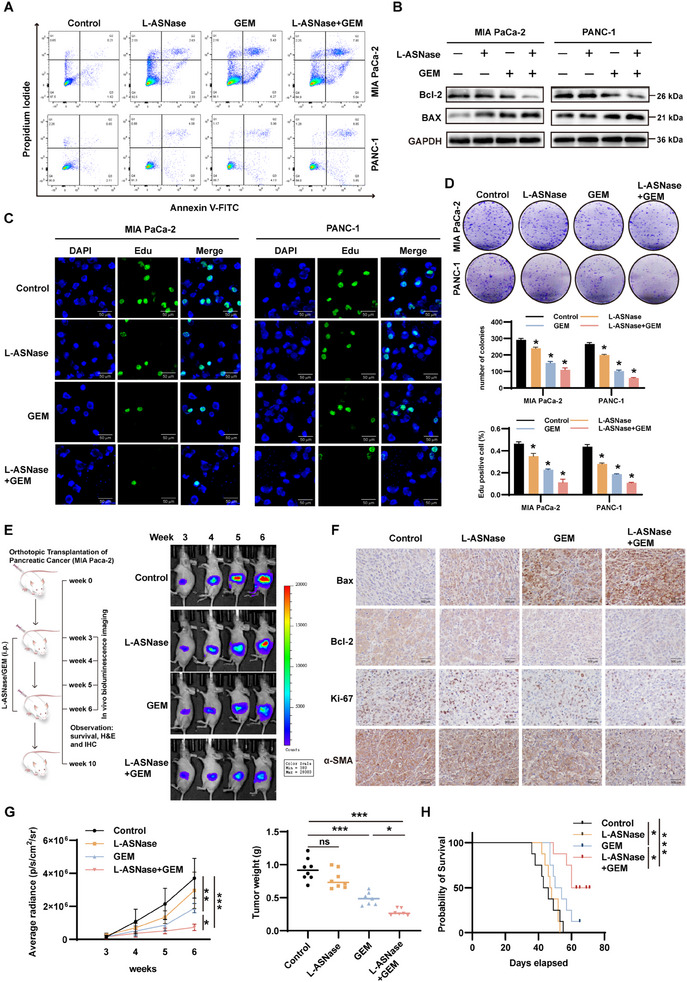
Validation of GEM combined with L‐ASNase to enhance the therapeutic effect of pancreatic cancer in vivo and in vitro. A) Flow cytometry showed that L‐ASNase combined with GEM promoted apoptosis of pancreatic cancer cells more significantly compared with control, L‐ASNase, and GEM groups. B) Western blot detected the apoptosis of pancreatic cancer cells in different drug treatments. C‐D) EdU immunofluorescence and clone formation assays showed the inhibition of pancreatic cancer cell proliferation when treated with different drugs. E) Flowchart of MIA PaCa‐2 luciferase orthotopic mouse model construction and drug injection (left) as well as representative bioluminescence images of pancreatic cancer mice at different times after drug administration (right). F) Immunohistochemistry of Ki‐67, Bax, Bcl‐2, and α‐SMA in tumors of pancreatic cancer mice in different treatment groups. G) Curve of average radiation fluctuation with time and tumor weight of mice. H) Survival curves of mice with pancreatic cancer at different drug treatments; ^*^ *p* < 0.05; ^**^ *p* < 0.01; ^***^ *p* < 0.001; ns: not significant.

As Asn promoted the activation of PSCs and thus enhanced the resistance of pancreatic cancer cells to GEM, we further explored the effects of L‐ASNase and GEM on the growth of pancreatic cancer cells themselves. Flow cytometry and western blot analysis of apoptosis‐related proteins demonstrated the ability of both L‐ASNase and GEM to promote apoptosis compared with the control group, but the effect of L‐ASNase was significantly weaker, whereas L‐ASNase combined with GEM promoted apoptosis of pancreatic cancer cells (**Figure** **7**A,B; Figure S7C, Supporting Information). Meanwhile, the effects of different drug treatments on the proliferative ability of pancreatic cancer cells were also carried out. EdU proliferation and clone formation assays showed that L‐ASNase combined with GEM had a stronger ability to inhibit proliferation than the control or single drug use (Figure 7C,D). In addition, We obtained the synergy scores of the two drugs in pancreatic cancer cells through measuring the inhibitory effects of drug combinations with different concentrations of L‐ASNase and GEM, and the results showed that the synergy scores of MIA PaCa‐2 and PANC‐1 were 4.419 and 5.028, respectively, suggesting the additive effect of the two drug combinations (Figure S7D, Supporting Information).^[^ ^22^ ^]^

An orthotopic transplant tumor mouse model was used to evaluate the therapeutic effect of L‐ASNase combined with GEM compared to that of the control or monotherapy on pancreatic cancer. The experimental process is shown in Figure 7E‐left. Small animal live imaging was performed at the indicated times after the in situ injection of MIA PaCa‐2 cells into the pancreas, and it was observed that the combination of L‐ASNase and GEM significantly slowed tumor growth compared to the other groups (Figure 7E‐right). Immunohistochemical staining of mouse tumors showed that the combination of the two drugs promoted apoptosis reduced the proliferation of pancreatic cancer cells, and decreased the activation of PSCs, as indicated by the reduced expression of the activation marker α‐SMA and lower levels of fibrotic proteins such as collagen‐1 and fibronectin. (Figure 7F; Figure S7E, Supporting Information), which was consistent with the average radiation and the weight and volume of mouse tumors (Figure 7G; Figure S7F, Supporting Information). To better assess the effect of GEM in combination with or without L‐ASNase on the survival of mice with pancreatic cancer, we observed the mice for 10 weeks. Kaplan–Meier analysis showed that the L‐ASNase + GEM group had longer overall survival (OS) than the control, L‐ASNase alone, and GEM alone groups (Figure 7H).

## 3 Discussion

**Figure 8:**
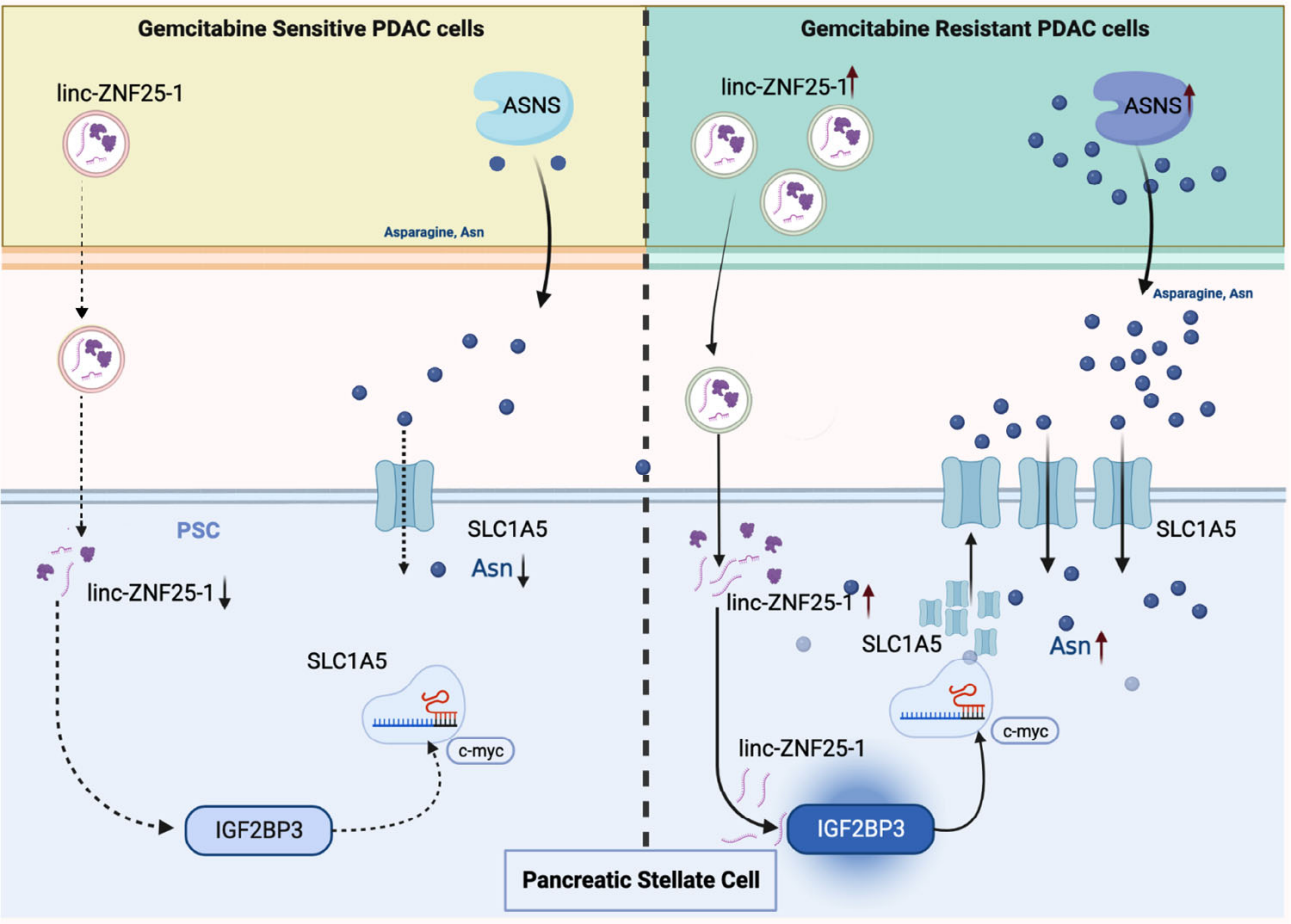
Mechanism diagram illustrating extracellular vesicle‐packaged linc‐ZNF25‐1 from pancreatic cancer cells promoting the uptake of Asn by PSCs.

GEM is one of the most important drugs for the treatment of advanced pancreatic cancer, and its effectiveness is constrained by the development of drug resistance.^[^ ^23^ ^]^ While PSCs have traditionally been considered a significant source of CAFs in pancreatic cancer, recent studies suggest that they contribute relatively minimally to the CAF population.^[^ ^24^ ^]^ However, activated PSCs are still important as they promote GEM resistance and facilitate the formation of a fibrous extracellular matrix, particularly through their role in ECM deposition.^[^ ^25^ ^]^ In this study, we compared the effects of parental and GEM‐resistant pancreatic cancer‐derived EVs on PSCs and analyzed the metabolites of EVs, and found that GEM‐resistant pancreatic cancer cells were able to produce and release more Asn, which facilitated the activation of PSCs and the production of extracellular matrix proteins. Based on lncRNA sequencing data of EVs, we confirmed that EVs derived from pancreatic cancer cells mediate the movement of linc‐ZNF25‐1 into PSCs and promote the uptake of Asn through the IGF2BP3/c‐Myc/SLC1A5 pathway (**Figure** **8**). An in vivo study elucidated the pro‐oncogenic effects of linc‐ZNF25‐1 and L‐ASNase combined with GEM to enhance their therapeutic effects in pancreatic cancer.

Unlike other malignant tumors, pancreatic cancer is rich in mesenchymal components. The interaction between pancreatic cancer cells and the tumor microenvironment is the main driver of fibrous stroma formation.^[^ ^4^ ^]^ Dense fibrous connective tissue not only forms a physical barrier but also interacts with pancreatic cancer cells to promote GEM resistance.^[^ ^26^ ^]^ EVs are important components of signaling between tumor cells and cells in the tumor microenvironment.^[^ ^27^ ^]^ Our study demonstrated the ability of pancreatic cancer cell‐derived EVs to activate PSCs and induce the expression of Collagen‐1, Fibronectin, and that EVs from GEM‐resistant pancreatic cancer cells had more significant effects. Similarly, pancreatic cancer cell‐derived EVs carrying IL‐17B can activate PSCs and induce IL‐17RB expression, thereby increasing oxidative phosphorylation and activating tumor cells in a feedback loop.^[^ ^28^ ^]^ Furthermore, in non‐solid tumors, such as leukemia, EVs secreted by leukemic cells can remodel the tumor microenvironment by promoting CAF activation, thereby making it more suitable for tumor cell growth and drug resistance.^[^ ^29^ ^]^

Glucose metabolism in tumors is considered one of the most important metabolic alterations in cancer cells,^[^ ^30^ ^]^ and amino acids are important nutrients that support cancer cell metabolism.^[^ ^31^ ^]^ Glutamine is the most abundant free amino acid in the body and serves as a carbon and nitrogen source to support cancer cell biosynthesis and energy metabolism.^[^ ^32^ ^]^ It was found that pancreatic ductal adenocarcinoma cells in a nutrient‐poor microenvironment were able to promote autophagy in PSCs and, thus, secrete alanine. Alanine, then, enters pancreatic cancer cells to participate in the tricarboxylic acid cycle, which promotes cancer cell proliferation.^[^ ^33^ ^]^ Additionally, serine and glycine facilitate nucleotide biosynthesis and DNA repair, supporting rapid cancer cell growth.^[^ ^34^ ^]^ The use of asparagine has also led to the use of L‐ASNase to treat leukemia by supporting the growth of tumor cells.^[^ ^8^ ^]^ Beyond the metabolic alterations inherent in cancer cells, metabolic alterations in TME cells may contribute to cancer progression. Our study found that GEM‐resistant pancreatic cancer cells have elevated ASNS expression and can synthesize and release more Asn, which promotes PSC activation and fibrous stroma formation. Additionally, it supports proliferation under limited respiration, with excess production driven by GCN2 activation.^[^ ^35^ ^]^ Wu et al. also found that elevated Asn levels enhanced the activation of CD8+ T cells and affected tumor cells in vitro and in vivo, whereas restriction of dietary Asn, ASNase administration, or inhibition of the Asn transporter protein SLC1A5 impaired CD8+ T cell activity and response.^[^ ^20^ ^]^ Thus, to understand how tumor cells reprogram their microenvironment to influence tumor growth requires further investigation.

EVs are important mediators of intercellular communication, mediating the exchange of information between cells by carrying substances including proteins, nucleic acids, lipids, and metabolites.^[^ ^12^, ^36^ ^]^ Among these, lncRNAs in EVs play a crucial role in regulating processes such as cancer proliferation, metastasis, and chemoresistance.^[^ ^37^ ^]^ In this study, we demonstrated that linc‐ZNF25‐1 was released from pancreatic cancer cells into PSCs via EVs. Mechanistically, linc‐ZNF25‐1 directly interacts with IGF2BP3 in PSCs and promotes nuclear ectopia of IGF2BP3. IGF2BP3 is an m^6^A reading protein that recognizes and binds to m^6^A methylation‐rich sites. c‐Myc is a classical target of IGF2BP3 and a regulator of a variety of transporter proteins.^[^ ^21a^ ^]^ Here, we demonstrated that IGF2BP3 was able to stabilize c‐Myc mRNA by binding to the CRD region of c‐Myc mRNA, which upregulated the expression of SLC1A5 in PSCs, ultimately leading to an increase in the uptake of Asn by PSCs via SLC1A5. In vitro and in vivo studies have confirmed that pancreatic cancer cells overexpressing linc‐ZNF25‐1 exhibit greater proliferation and drug resistance. Our study suggests that extracellular vesicular lncRNAs can mediate metabolic reprogramming between cancer and stromal cells. However, the exploration of potential therapeutic targets based on these lncRNAs requires further investigation. Additionally, future studies could employ broader transcriptomic approaches, such as whole transcriptome sequencing, to gain a more comprehensive understanding of the RNA content in extracellular vesicles.

The TME is not only a place for cancer cells to survive but also a major cause of chemotherapy resistance.^[^ ^38^ ^]^ In pancreatic cancer, an abundant mesenchymal component is favorable for GEM resistance. Our study showed that Asn can activate PSCs to promote the formation of fibrous stroma. Therefore, targeting the metabolism of stromal cells can be used to sensitize pancreatic cancer cells to the therapeutic effects of existing drugs such as GEM. In the present study, L‐ASNase was used to eliminate Asn. L‐ASNase degrades Asn in the pancreatic cancer mesenchyme, leading to inhibition of PSCs activation and suppression of fibrosis, therefore sensitizing the anti‐pancreatic cancer effect of GEM. Mouse models of in situ transplantation of pancreatic cancer demonstrated that L‐ASNase alone does not significantly inhibit the proliferation of pancreatic cancer. In pancreatic cancer cells, although L‐ASNase combined with GEM had better therapeutic effects than single drug application, the drug combination analysis only showed the additive effect of these two drugs. In contrast, in the mouse in situ pancreatic cancer model, L‐ASNase combined with GEM had more significant efficacy than GEM or L‐ASNase alone and was able to significantly prolong the OS of the mice, which may be attributed to the fact that L‐ASNase inhibited the activation of PSCs in the pancreas of the mice and promoted the therapeutic effect of GEM.

Asn is currently the most common target in amino acid depletion therapies, especially in acute lymphoblastic leukemia, where the introduction of ASNase greatly increases the cure rate.^[^ ^8^ ^]^ In contrast, previous strategies using ASNase in other tumor therapies did not show significant results, possibly because of the existence of signaling pathways that induce ASNS expression.^[^ ^39^ ^]^ Asparagine restriction in melanoma and pancreatic cancer cells has been shown to activate MAPK signaling, ultimately leading to enhanced translation of activating transcription factor 4 (ATF4) mRNA and expression of ASNS; MAPK inhibition sensitizes melanoma and pancreatic cancer cells to asparagine restriction.^[^ ^10^ ^]^ In pancreatic cancer, L‐ASNase not only targets Asn but also exhibits a crucial glutaminase co‐activity, which is linked to KRAS mutations. Research has identified glutamine synthetase (GS) as a marker of resistance to L‐ASNase, suggesting potential avenues for targeted therapies that could overcome this resistance.^[^ ^40^ ^]^ Further studies are needed to explore how modulating GS activity could enhance the efficacy of L‐ASNase treatment. Eryaspase is an ASNase encapsulated in red blood cells, and the results of a second‐line therapy evaluating Eryaspase in combination with chemotherapy for the treatment of advanced pancreatic cancer showed that Eryaspase improved its survival rate.^[^ ^41^ ^]^ Consequently, targeting the metabolism to sensitize the therapeutic effects of existing drugs is possible.

This study primarily utilized immunocompromised in vivo models, which presents an opportunity for further exploration into how the identified cross‐talk between pancreatic cancer and PSCs may interact with immune cell populations. We acknowledge that considering the immune tumor microenvironment could enrich our understanding of tumor dynamics and treatment responses. Future investigations that incorporate immunocompetent models may provide valuable insights into these interactions.

In conclusion, this study demonstrated that pancreatic cancer cell‐derived EVs facilitate Asn uptake and activation in PSCs by upregulating proteins that transport Asn, thereby favoring GEM resistance in pancreatic cancer. L‐ASNase, which eliminates Asn, is more effective in combination with GEM for the treatment of pancreatic cancer. Our study revealed a potential therapeutic strategy to target the metabolism of the tumor microenvironment, thereby sensitizing chemotherapeutic agents.

## 4 Experimental Section

### Cell Culture

The human‐derived pancreatic cancer cell lines MIA PaCa‐2 and PANC‐1 used in this study were purchased from the American Type Culture Collection (ATCC, Manassas, USA) and identified by short tandem repeat mapping (Wuhan, China), while the GEM‐resistant pancreatic cancer cell lines MIA PaCa‐2^R^ and PANC‐1^R^ were constructed by the group.^[^ ^16^ ^]^ All cells were cultured in Dulbecco's Modified Eagle medium (DMEM) (Gibco, USA), supplemented with 10% fetal bovine serum (FBS) and 1% penicillin/streptomycin and placed in an incubator at 37 °C with 5% CO_2_.

### Extraction, Characterization, and Internalization of EVs

EVs (derived from MIA PaCa‐2/ MIA PaCa‐2^R^ and PANC‐ 1/ PANC‐1^R^, referred to as EV^m^/ EV^mR^ and EV^P^/ EV^PR^, respectively) were identified after extraction by differential ultracentrifugation. The EV concentration was determined using the BCA method. The obtained EVs were labeled with PKH26 (PKH26GL, Sigma‐Aldrich, USA) and then added to PSCs, and the uptake of EVs by PSCs was observed by immunofluorescence. Detailed methods can be found in the Supplementary material and methods.

### RNA Interference and Plasmid Transfection

The sequence of the small interfering RNA (siRNA) is shown in Table S1 (Supporting Information). Transient transfection was performed with Lipofectamine 3000 (Invitrogen, New York, USA), according to the instructions. Lentiviral‐mediated stable transfection was performed according to the manufacturer's protocol.

### RNA Extraction, Reverse Transcription and qRT‐PCR

Total RNA was extracted from cells using the RNA Purification Kit (B0004D, EZBioscience, China) and from tissues using the RNA Purification Kit Plus (EZB‐RN001‐plus, EZBioscience). For RNA extraction from EVs, the Exosome RNA Purification Kit (EZB‐exo‐RN1, EZBioscience) was used, following the manufacturer's instructions. The concentration and purity of RNA were measured using a spectrophotometer. Total RNA was reverse transcribed to cDNA using PrimeScript RT Master Mix (TaKaRa Bio, Japan), and quantitative PCR was performed using SYBR Green PCR Master Mix (Vazyme Biotech, China) with GAPDH as the control gene. Relative mRNA expression was estimated using the 2^−ΔΔCt^ method. The primer sequences are listed in Table S2 (Supporting Information).

### Western Blotting

Cells were lysed using RIPA lysis buffer to isolate total protein, and the protein concentration was determined using a bicinchoninic acid (BCA) kit. Protein samples (20 µg) were separated by 7.5% or 10% SDS‐PAGE and transferred onto polyvinylidene fluoride membranes. After being closed with 5% BSA for 1 h at 37 °C, the membranes were incubated with the indicated primary antibodies at 4 °C overnight. After washing, membranes were incubated with secondary antibodies for 1 h at room temperature. Protein bands were observed using ECL reagent after washing again. The primary antibodies used are listed in Table S3 (Supporting Information).

### Immunofluorescence

The cells were seeded onto 12 well plates with glass slides and cultured overnight. The next day, slides moistened with PBS were fixed with 4% paraformaldehyde, permeabilized with 0.5% Triton X‐100, and blocked with 5% goat serum. Specific primary antibodies were, then, added and the mixture was incubated at 4 °C overnight. After incubation with a fluorescent secondary antibody and DAPI re‐staining of nuclei, the slides were sealed, and representative images were observed under a fluorescence microscope (Olympus BX63, Japan).

### Immunohistochemistry (IHC)

After dewaxing and hydration, the sections were heated in citrate or EDTA buffer in a microwave oven for 20 min to repair the antigens. The slides were pretreated with 3% H_2_O_2_ for 10 min at room temperature and sealed with 5% goat serum for 1 h at room temperature. The slides were washed with PBST and incubated with primary antibody at 4 ° C overnight. On the second day after cleaning with PBST, the chemiluminescence reporter enzyme horseradish peroxidase (HRP) – labeled secondary antibody was added for incubation. After washing, the slides were developed with DAB and stained with hematoxylin. Finally, the images were captured using a microscope.

### Cell Viability Assay

To determine the effect of different concentrations of Asn on PSC growth, 5000 cells per well were seeded in a 96‐well plate. The cells were treated with various concentrations of Asn (166.67, 250, and 500 µM) and incubated for 5 consecutive days. Cell viability was assessed daily using the CCK8 assay. On each day, 10 µL of CCK8 reagent was added to each well, and the plate was incubated for 2 h at 37 °C. Absorbance was measured at 450 nm using a microplate reader to evaluate cell viability.

### Asparagine Assay

The asparagine content was measured using an asparagine content kit (Grace Biotechnology, China) following the manufacturer's instructions. In brief, asparagine was decomposed into aspartate and NH_4_^+^ by asparaginase, after which glutamate dehydrogenase reacted the NH_4_^+^ with α‐ketoglutarate and oxidized NADH; the amount of asparagine (Asn) was calculated by measuring the decrease in NADH at the specific absorption wavelength of 340 nm.

### ^15^N‐Asn Quantification and LC‐MS Analysis

PSCs were seeded in 6‐cm dishes and incubated overnight. The next day, cells were washed twice with PBS and incubated in a medium containing 250 µm ^15^N‐asparagine for 24 h. The metabolites in the cells and supernatants were extracted using an appropriate amount of mass spectrometry‐grade methanol for LC‐MS analysis.

### Nucleocytoplasmic Separation

Nucleocytoplasmic separation was performed using NE‐PER Nuclear and Cytoplasmic Extraction Reagent (78 833, Thermo Fisher Scientific, USA). Then, qRT‐PCR was performed to detect the expression levels of GAPDH, U6, and linc‐ZNF25‐1 in the cytoplasm and nucleus of pancreatic cancer cells.

### Construction of PAAD Tissue Chip

Discarded pancreatic tissues and tumor specimens were collected from patients admitted to Sun Yat‐Sen Memorial Hospital with histopathological and clinical diagnosis of PAAD from 2013 to 2021. Clinical and follow‐up information of these patients was also collected for disease analysis only. All samples were obtained from discarded samples after surgical resection and approved by the Institutional Ethics Committee of Sun Yat‐Sen Memorial Hospital (SYSKY‐2023‐491‐01).

### RNA In Situ Hybridization

Paraffin‐embedded tissue sections were deparaffinized and rehydrated through a series of xylene and graded ethanol washes, followed by antigen retrieval in 1× retrieval solution. The sections were then digested with Proteinase K and treated with 3% hydrogen peroxide to block endogenous peroxidase activity. Pre‐hybridization was performed at 40 °C, followed by hybridization with target probe 1 and target probe 2, with washing in graded SSC solutions after each step. DIG‐labeled signal probe hybridization was carried out at 37 °C, followed by additional washes. The sections were then blocked and incubated with anti‐DIG (HRP) antibody, developed with DAB, counterstained with hematoxylin, dehydrated, and mounted.

### Bioinformatics Analysis

Possible binding proteins of linc‐ZNF25‐1 were predicted using (http://s.tartaglialab.com/page/catrapid_omics2_group). Based on the minimum free energy (MFE) and the partition function, the secondary structure of lincZNF25‐1 was established using (http://rna.tbi.univie.ac.at/cgi‐bin/RNAWebSuite/RNAfold.cgi).

### RNA Immunoprecipitation and Pull‐Down Analysis

RNA immunoprecipitation was performed using the RNA Binding Protein Immunoprecipitation Kit (Giese Biotech, China), and RNA pull‐down was performed using the PureBinding® RNA‐Protein pull‐down kit (Giese Biotech, China) according to the manufacturer's instructions.

### mRNA Stability Assay

To calculate the half‐life of c‐Myc mRNA, control or IGF2BP3 knockout PSCs were seeded in 6‐well plates and allowed to reach 50% confluence after 24 h. The cells were then treated with 5 µg/ml actinomycin D and collected at the indicated time points. Total RNA was extracted and analyzed by qRT‐PCR using a previously described method. The RNA concentration decay over time followed an exponential pattern and the decay constant 𝑘. 𝑘 was determined by comparing the concentrations at different time points. The half‐life was then estimated based on the rate of decay.

### IC50

Pancreatic cancer cells from the different treatment groups were inoculated into 96‐well plates at 1 × 10^4^ cells per well. After cell adhesion, different drug concentrations were added to each group. After 72 h of cell incubation, the cells were rinsed with PBS and cell viability was determined using the CCK‐8 (APExBIO, USA) reagent; OD450 was measured spectrophotometrically 2 h after the addition of the CCK‐8 reagent.

### EdU Proliferation Assay

EdU staining was performed using the EdU Cell Proliferation Kit (UELandy, China) according to the manufacturer's instructions. Cells were incubated with 10 µm EdU for 2 h. After rinsing with PBS, the cells were fixed with 4% paraformaldehyde, permeabilized with 0.5% Triton X‐100, and treated with a Click‐iT working solution for 30 min. Finally, after incubation with a DAPI solution, the cells were observed under a fluorescence microscope (Olympus BX63, Japan).

### Colony Formation Assay

Five hundred cells per well were inoculated into 6‐well plates and cultured for 2 weeks. Cells were fixed with 4% paraformaldehyde and stained with 0.4% crystal violet. Finally, plate colonies were photographed and counted.

### Flow cytometry

Apoptosis was detected by flow cytometry using an Annexin V‐PI Apoptosis Detection Kit (eBioscience, USA). All adherent and floating cells were collected and rinsed with PBS; subsequently, the cells were treated with Annexin V‐FITC and propidium iodide under light‐avoidance conditions and then analyzed instantly with the FACS Calibur system (BD Biosciences).

### Establishment of Orthotopic Transplanted Tumor of Human Pancreatic Cancer in Mice

Orthotopic transplantation of human pancreatic cancer in mice was used to evaluate the role of linc‐ZNF25‐1 in pancreatic cancer and the combined effect of L‐ASNase and GEM on chemoresistance. The process of the orthotopic transplantation tumor model was consistent with that described in the previous study.^[^ ^16^ ^]^ All mouse studies were authorized by the Animal Care and Use Committee of Sun Yat‐Sen University (SYSU‐IACUC‐2024‐000245) and followed the National Institutes of Health Guide for the Care and Use of Laboratory Animals. Experimental animal studies were conducted in accordance with relevant ethical standards for animal research.

### Data Analysis

Data were presented as mean ± standard deviation for normally distributed variables and as median (interquartile range) for non‐normally distributed variables.

Differences between groups were analyzed using the unpaired two‐tailed Student's *t*‐test or the One‐way ANOVA. For comparisons between groups of categorical data, the Chi‐square test or Fisher's exact test was used as appropriate. Survival curves were generated using the Kaplan‐Meier method. All experiments were independently repeated three times. A *P* value of <0.05 was considered statistically significant.

### Others

Methodological details and other methods and materials can be found in supplementary methods and materials in the Supporting Information.

### Ethics Approval Statement

This study was approved by the Medical Ethics Committee of Sun Yat‐Sen Memorial Hospital of Sun Yat‐Sen University (approval number: SYSKY‐2023‐491‐01). The requirement for informed consent was waived, as the study involved the use of de‐identified data/samples and posed minimal risk to participants.

## Conflict of Interest

The authors declare no conflict of interest.

## Supporting information



Supporting Information

## Data Availability

The data that support the findings of this study are available from the corresponding author upon reasonable request.
