# Supplementary material for: Extracellular Vesicle‐Packaged Linc‐ZNF25‐1 from Pancreatic Cancer Cell Promotes Pancreatic Stellate Cell Uptake of Asparagine to Advance Chemoresistance
Source: Adv Sci (Weinh). 2025 Mar 5;12(16):2413439. doi: 10.1002/advs.202413439 (PMC12021039; doi:10.1002/advs.202413439)
Supplement: Supplementary file 1 — Supporting Information [file ADVS-12-2413439-s001.docx]

Supporting Information

Extracellular Vesicle-packaged Linc-ZNF25-1 from Pancreatic Cancer Cell Promotes Pancreatic Stellate Cell Uptake of Asparagine to Advance Chemoresistance

Miao Yu, Mingxin Su, Zhenfeng Tian, Lele Pan, Zongmeng Li, Enlai Huang, Yinting Chen*

**Supplementary Figures and Tables**


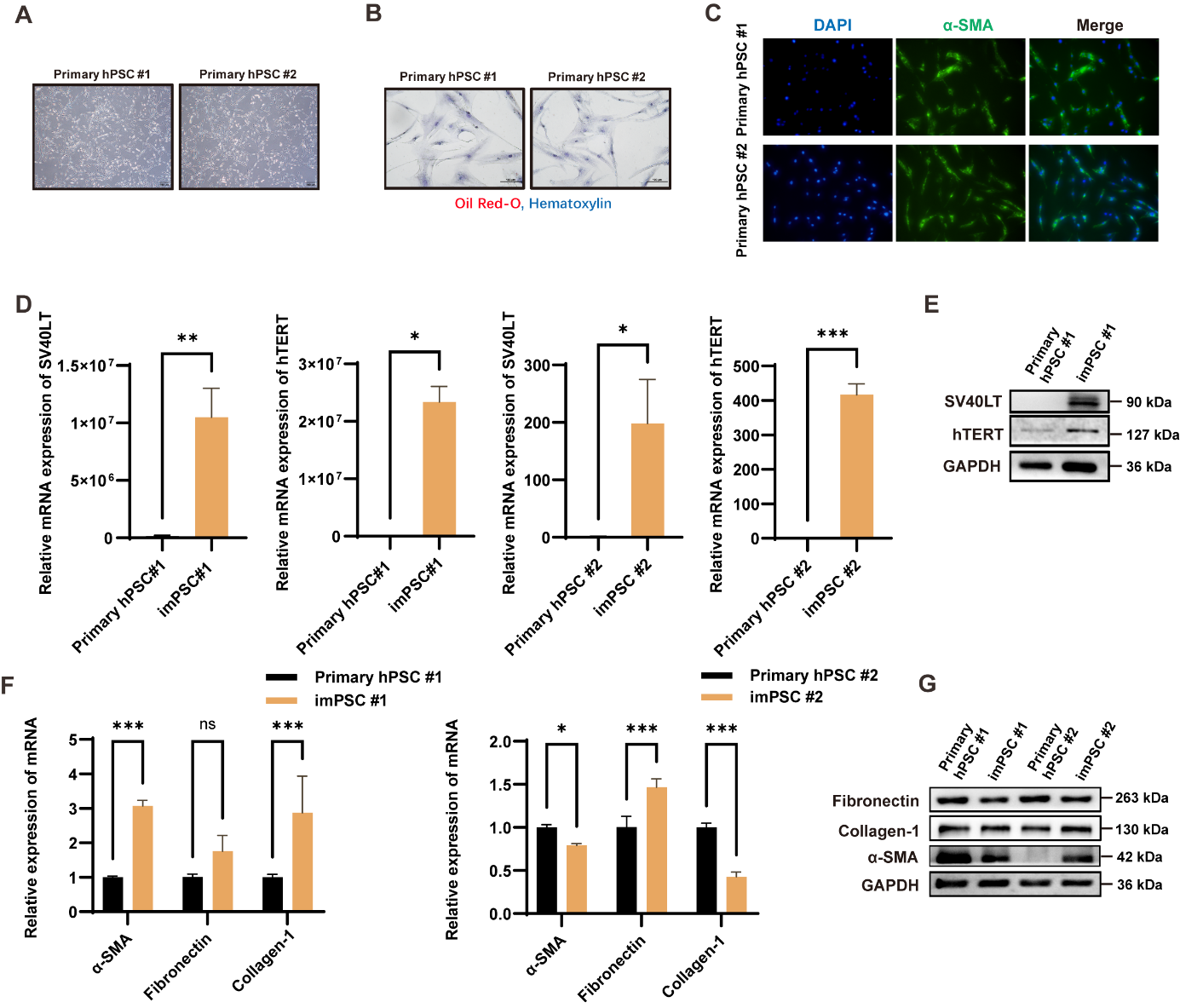


**Figure S1**. Primary culture and immortalization of PSCs. A) Light microscope images of primary PSCs. B) Oil red O staining of primary PSCs. C) Immunofluorescence of α-SMA in primary PSCs. D-E) qRT-PCR and Western blot to detect the expression of SV40LT and hTERT in primary and immortalized PSCs. F-G) qRT-PCR and Western blot for the detection of α-SMA, Fibronectin and Collagen-1 in primary and immortalized PSCs; **P* < 0.05; ***P* < 0.01; ****P*< 0.001.


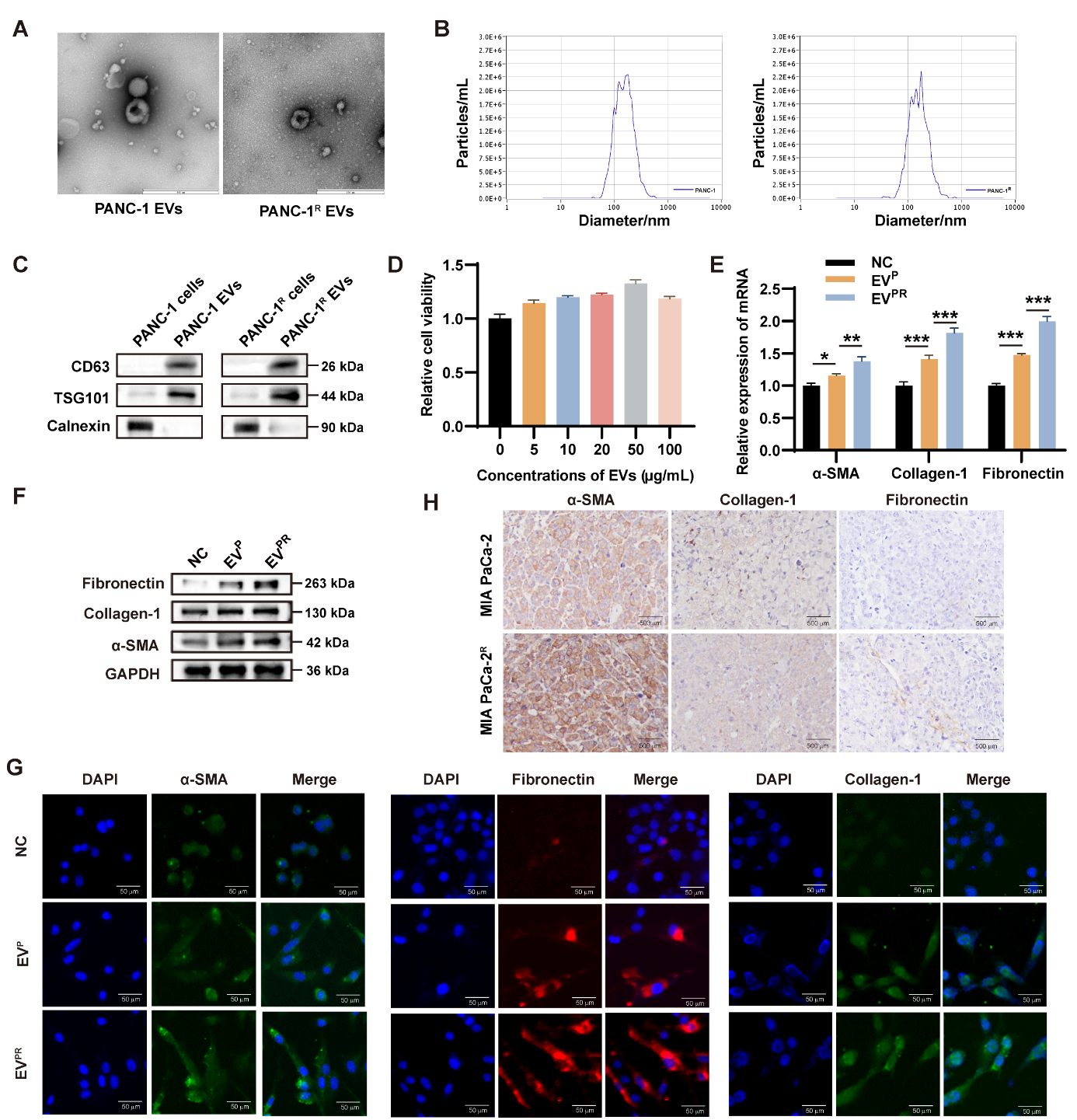


**Figure S2.** EVs derived from pancreatic cancer cells promote PSCs activation. A) TEM images of EVs isolated from PANC-1/PANC-1^R^. B) NTA analysis of EVs derived from PANC-1/PANC-1^R^. C) Western blot of EVs markers. D) Cell proliferation measured by CCK-8 assay after treatment of PSCs with different concentrations of EVs. E-G) mRNA and protein expression of α-SMA, Collagen-1, and Fibronectin during incubation of PSCs with PANC-1/PANC-1^R^-derived EVs and their immunofluorescence images; scale bar, 50 μm. H) Immunohistochemistry of α-SMA, Collagen-1 and Fibronectin was performed in mouse parental pancreatic tumors and GEM-resistant pancreatic tumors; **P* < 0.05; ***P* < 0.01; ****P*< 0.001.


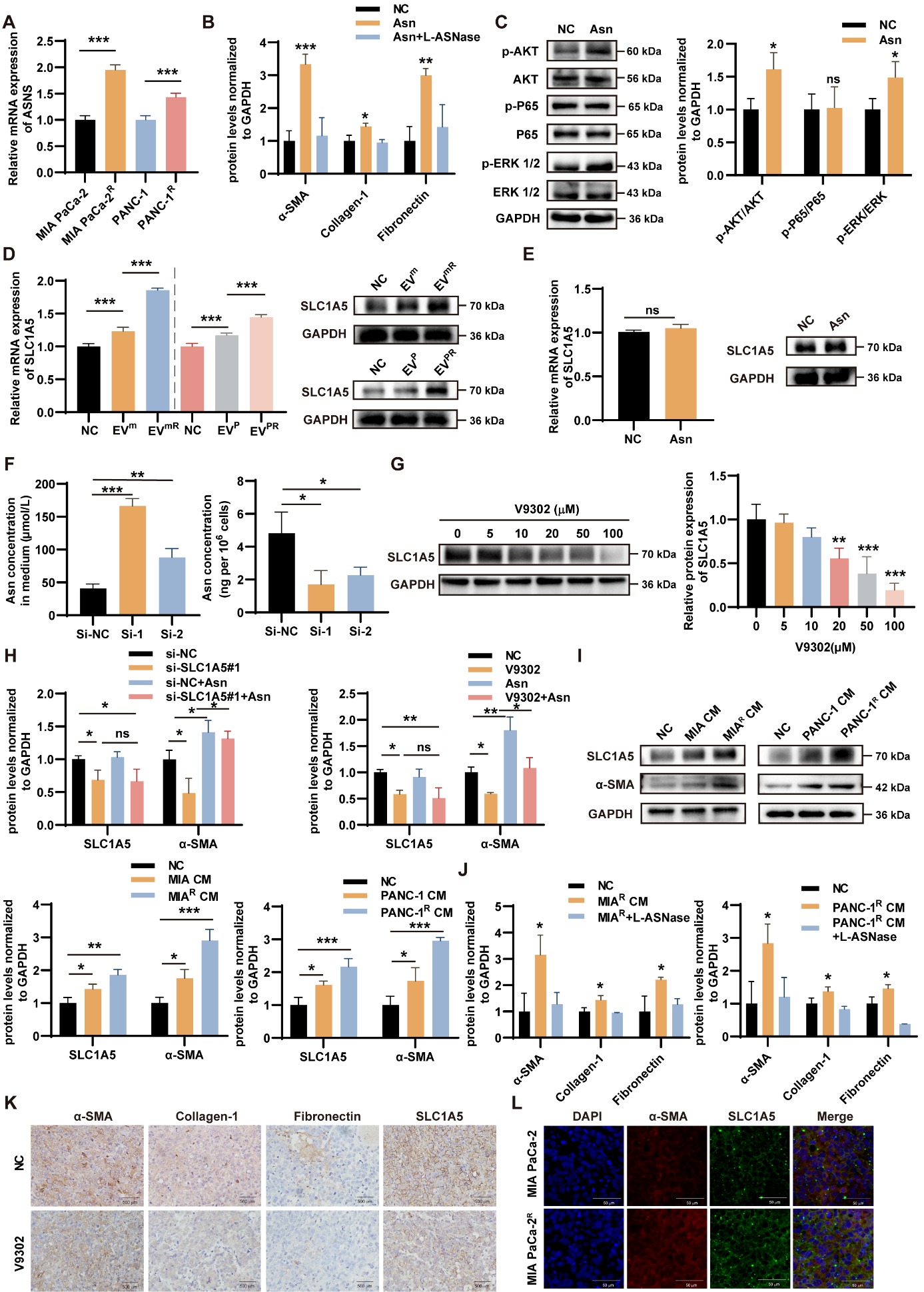


**Figure S3.** Enhanced activation of PSCs by SLC1A5 uptake of GEM-resistant pancreatic cancer cell-derived Asn. A) qRT-PCR to measure ASNS expression in MIA PaCa-2/MIA PaCa-2^R^ and PANC-1/PANC-1^R^ cells. B) Gray-scale analysis of western blot for the action of Asn with or without L-ASNase on α-SMA, Collagen-1, and Fibronectin in PSCs. C). Western blot analysis showing the activation of PSCs through the AKT and MAPK pathways upon Asn treatment. D) Pancreatic cancer parental and GEM-resistant cell-derived EVs up-regulate SLC1A5 expression in PSCs. E) Asn has no significant effect on SLC1A5 expression in PSCs. F) Effect of knockdown of SLC1A5 on the uptake of Asn by PSCs. G) Treatment with different concentrations of V9302 affects the expression of SLC1A5 in PSCs. H) Asn-induced α-SMA enhanced in PSCs can be reversed by si-SLCA5 and V9302. I) Conditioned medium for GEM-resistant pancreatic cancer cells promotes PSCs activation more. J) Effect on PSCs activation when cultured in GEM-resistant pancreatic cancer cell conditioned medium with or without L-ASNase. K) Immunohistochemistry of α-SMA, Collagen-1, Fibronectin and SLC1A5 of pancreatic cancer mice treated with PBS or V9302. L) Immunofluorescence for α-SMA and SLC1A5 in parental and GEM-resistant pancreatic tumors in mice; **P* < 0.05; ***P* < 0.01; ****P*< 0.001; ns: not significant.


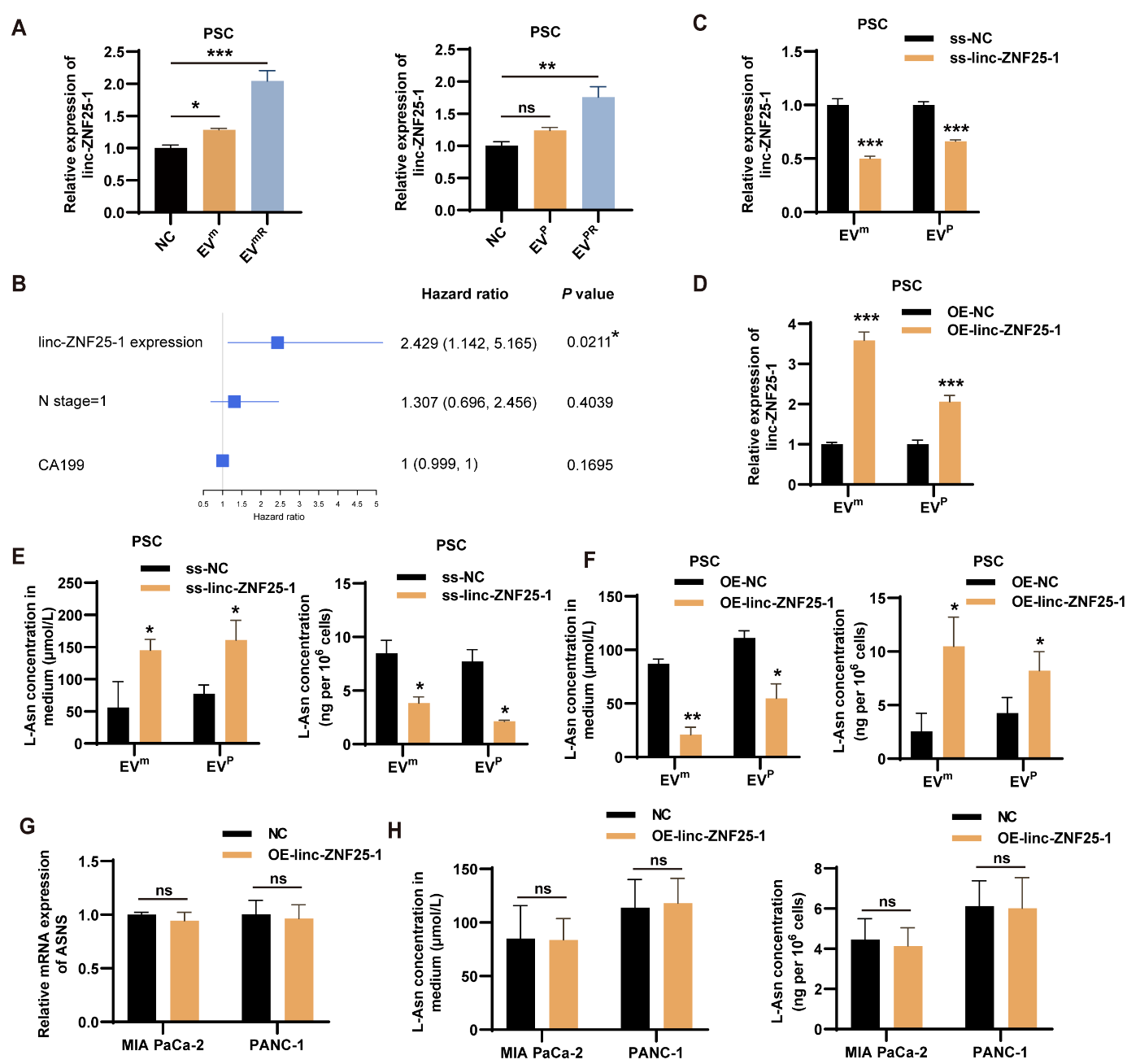


**Figure S4.** Upregulation of SLC1A5 in PSCs by linc-ZNF25-1 mediated by pancreatic cancer cell-derived EVs. A) Effects of parental and GEM-resistant pancreatic cancer cell-derived EVs on linc-ZNF25-1 levels in PSCs. B). Cox regression analysis was conducted to evaluate the impact of linc-ZNF25-1 expression on prognosis, with N stage and CA199 as covariates. C-D) Effects of silencing or overexpressing linc-ZNF25-1 pancreatic cancer cell-derived EVs on linc-ZNF25-1 levels in PSCs. E-F) Effect of silencing or overexpressing linc-ZNF25-1 pancreatic cancer cell-derived EVs on the ability of PSCs to uptake Asn. G) Expression of ASNS in pancreatic cancer cells overexpressing linc-ZNF25-1 detected by qRT-RCR. H) Concentration of Asn in pancreatic cancer cells overexpressing linc-ZNF25-1 and their conditioned medium; **P* < 0.05; ***P* < 0.01; ****P*< 0.001.


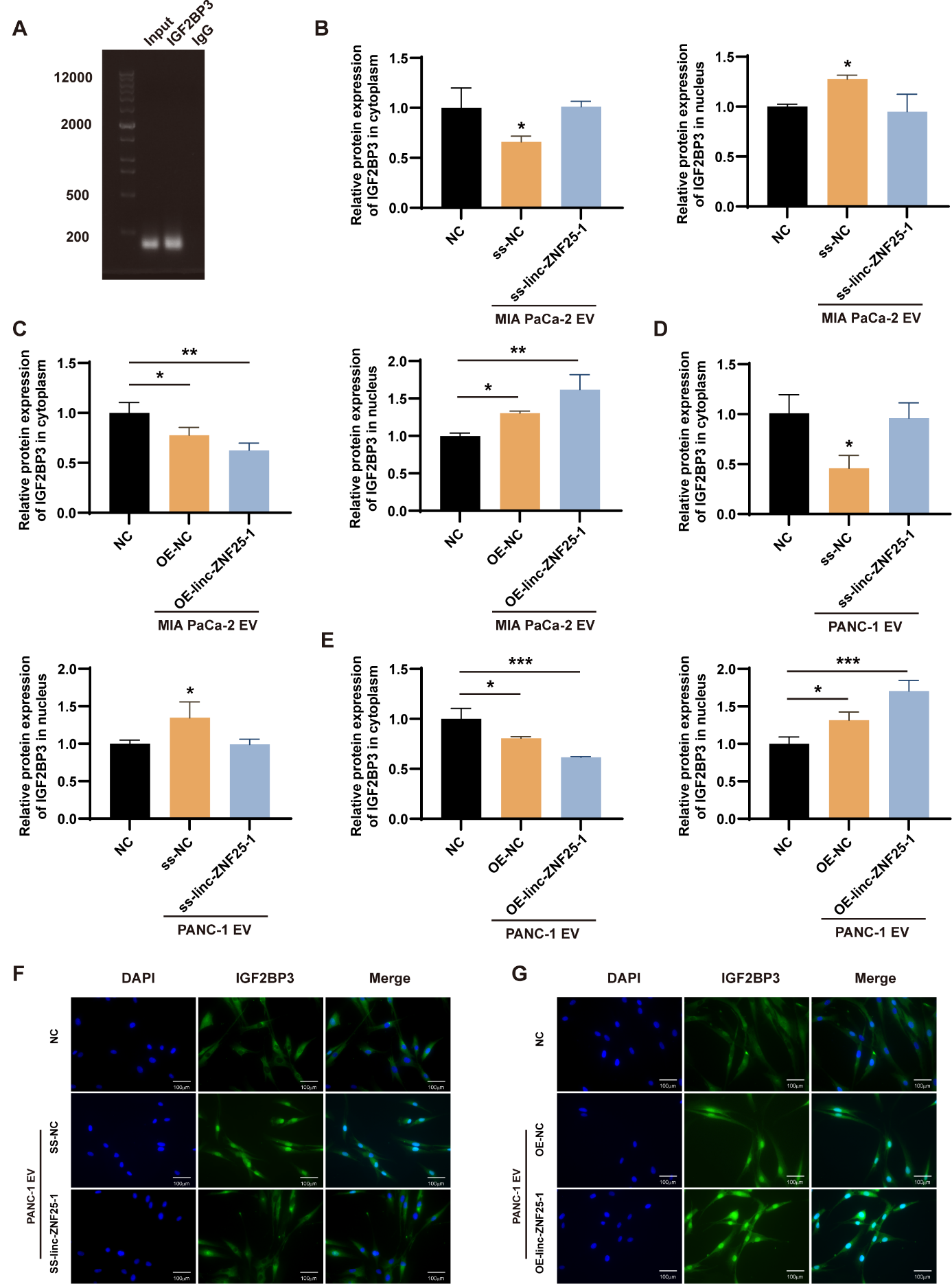


**Figure S5.** linc-ZNF25-1 binds IGF2BP3 and promotes IGF2BP3 nuclear translocation. A) Agarose gel plots of qRT-PCR products after RIP. B-C) Western blot analysis of IGF2BP3 after nucleoplasmic isolation of PSCs cells treated with different EVs of MIA PaCa-2. D-E) Western blot analysis of IGF2BP3 after nucleoplasmic isolation of PSCs cells treated with different EVs of PANC-1. F-G) Immunofluorescence staining shows changes in cytoplasmic and cytosolic localization of IGF2BP3 in PSCs cells treated with different EVs derived from PANC-1; **P* < 0.05; ***P* < 0.01; ****P*< 0.001.


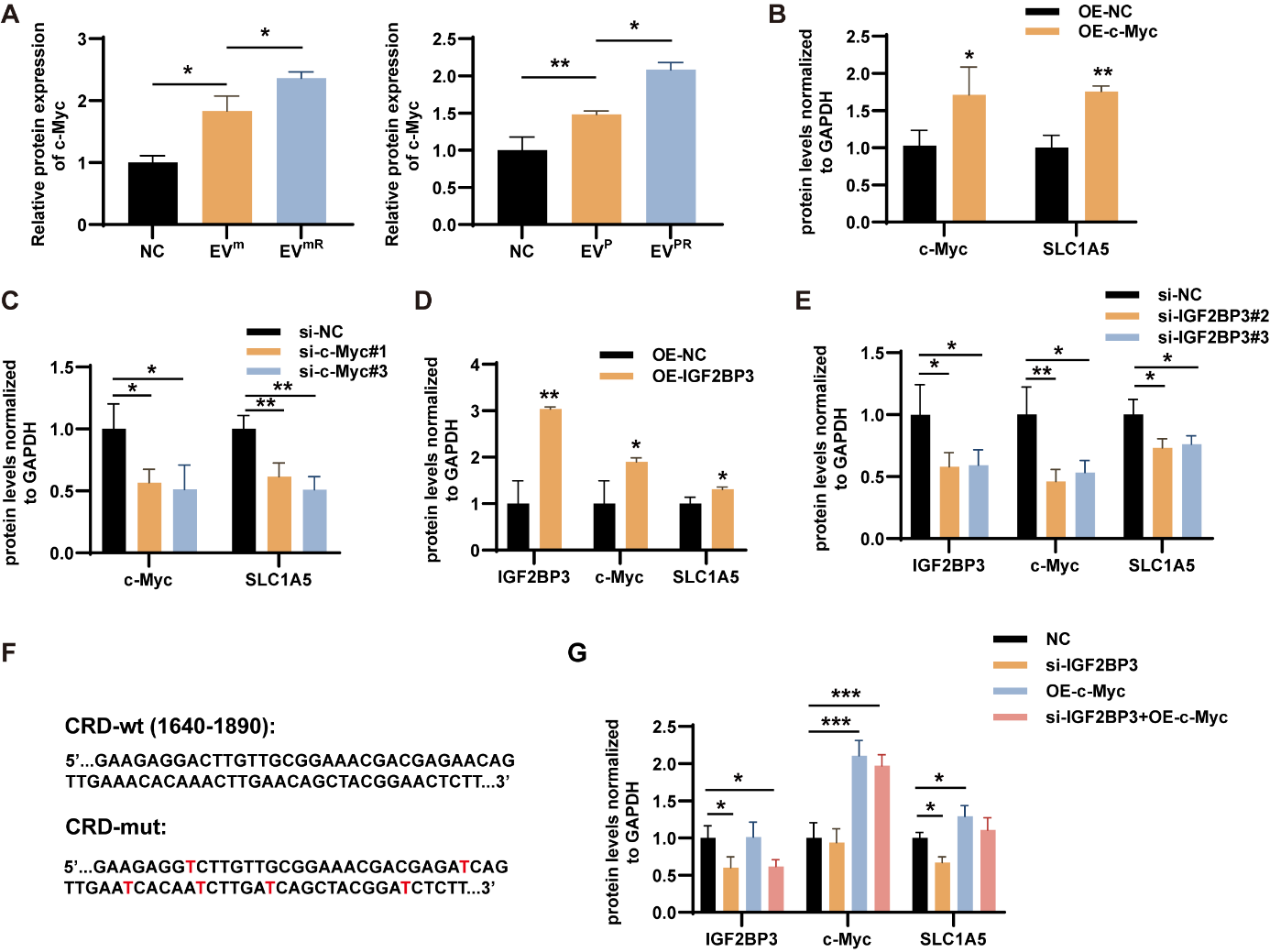


**Figure S6.** IGF2BP3 up-regulates SLC1A5 by stabilizing c-Myc mRNA. A) Grayscale analysis of c-Myc when parental or GEM-resistant pancreatic cancer cell-derived EVs acted on PSCs. B-C) Grayscale analysis of c-Myc and SLC1A5 when overexpression or silencing of c-Myc. D-E) Grayscale analysis of IGF2BP3, c-Myc and SLC1A5 when overexpression or silencing of IGF2BP3. F) Schematic representation of CRD-wt and CRD-mut in the c-Myc region bound to IGF2BP3. G) Gray scale analysis of SLC1A5 in PSCs with or without knockdown of IGF2BP3 and with or without overexpression of c-Myc treatment; **P* < 0.05; ***P* < 0.01; ****P*< 0.001.


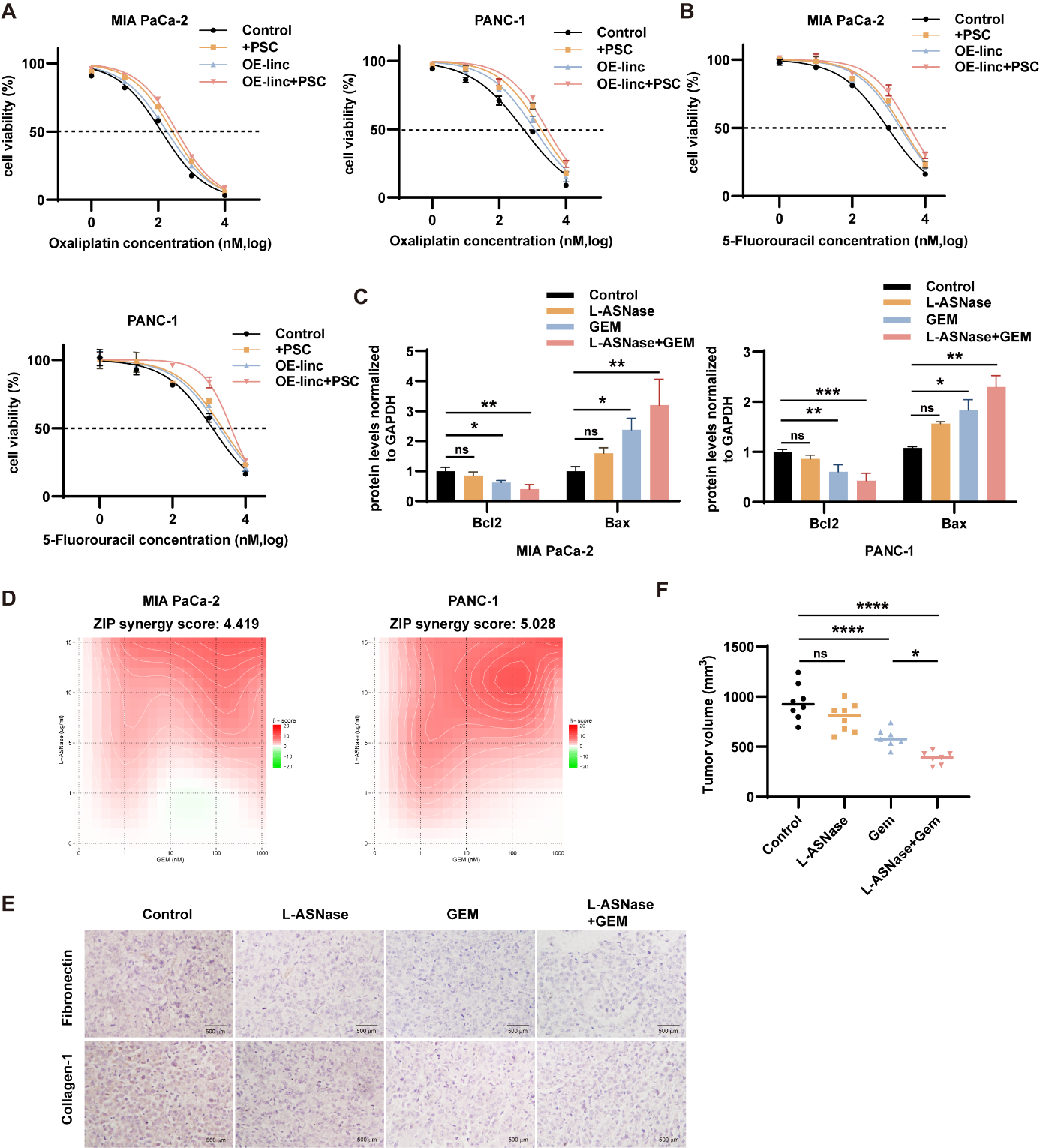


**Figure S7.** linc-ZNF25-1 promotes pancreatic cancer cell drug resistance and GEM combined with asparaginase to enhance the therapeutic effect of pancreatic cancer. A-B) Cell viability of pancreatic cancer cells with or without overexpression of linc-ZNF25-1 alone or co-cultured with PSCs after treatment with different concentrations of oxaliplatin or 5-fluorouracil for 72 h. C) Gray scale analysis of Bcl2 and Bax in pancreatic cancer cells treated with different drugs. D) Synergistic scores of L-ASNase and GEM combination in pancreatic cancer cells. E) Immunohistochemistry of Fibronectin and Collagen-1 in tumors of pancreatic cancer mice in different treatment groups. F) Tumor volume of mice in different treatment groups; **P* < 0.05; ***P* < 0.01; ****P*< 0.001; ns: not significant.

**Table S1.** The sequences of small interfering RNA

| **siRNA** | **Sequence (5’-3’)** |
| --- | --- |
| si-NC (sense) | UUCUCCGAACGUGUCACGUTT |
| si-NC (antisense) | ACGUGACACGUUCGGAGAATT |
| si-SLC1A5#1 (sense) | GCCUGAGUUGAUACAAGUGAATT |
| si-SLC1A5#1 (antisense) | UUCACUUGUAUCAACUCAGGCTT |
| si-SLC1A5#2 (sense) | CUGGAUUAUGAGGAAUGGAUATT |
| si-SLC1A5#2 (antisense) | UAUCCAUUCCUCAUAAUCCAGTT |
| si-IGF2BP3#1 (sense) | GGUGCUGGAUAGUUUACUATT |
| si-IGF2BP3#1 (antisense) | UAGUAAACUAUCCAGCACCTT |
| si-IGF2BP3#2 (sense) | GCUGAGAAGUCGAUUACUATT |
| si-IGF2BP3#2 (antisense) | UAGUAAUCGACUUCUCAGCTT |
| si-IGF2BP3#3 (sense) | GGACCAAGCUAGACAAGCATT |
| si-IGF2BP3#3 (antisense) | UGCUUGUCUAGCUUGGUCCTT |
| si-c-Myc#1 (sense) | GAGGAGACAUGGUGAACCATT |
| si-c-Myc#1 (antisense) | UGGUUCACCAUGUCUCCUCTT |
| si-c-Myc#2 (sense) | GCCACAGCAUACAUCCUGUTT |
| si-c-Myc#2 (antisense) | ACAGGAUGUAUGCUGUGGCTT |
| si-c-Myc#3 (sense) | CGAUGUUGUUUCUGUGGAATT |
| si-c-Myc#3 (antisense) | UUCCACAGAAACAACAUCGTT |

**Table S2.** Sequences of primers used in qRT-PCR

| **Primer** | **Forward (5’-3’)** | **Reverse (5’-3’)** |
| --- | --- | --- |
| SV40LT | GAAATGAGCCTTGGGACT | GGCTATGGGAATTGGAGT |
| h-TERT | GGCGACATGGAGAACAAGC | TCAGGGACACCTCGGACCAG |
| α-SMA | GTGTTGCCCCTGAAGAGCAT | GCTGGGACATTGAAAGTCTCA |
| Collagen-1 | GAACAGGGCGACAGAGGCATAAG | TCACGAACCACATTGGCATCATCAG |
| Fibronectin | TTGAGAAGAGTTACGAGTTGCCGATG | GCCACCGATCCAGACAGAGTATTTG |
| ASNS | GGAAGACAGCCCCGATTTACT | AGCACGAACTGTTGTAATGTCA |
| SLC1A5 | GCTGCTTATCCGCTTCTTC | TAAACCCACATCCTCCATCT |
| Linc-ZNF25-1 | GATAATTCCATTTGGGTCCATTCG | TGGAATCTTCCAATGGTCTCGA |
| c-Myc | TACAACACCCGAGCAAGGAC | GAGGCTGCTGGTTTTCCACT |
| IGF2BP3 | AGTTGTTGTCCCTCGTGACC | GTCCACTTTGCAGAGCCTTC |
| GAPDH | ATCACCATCTTCCAGGAGCGA | CCTTCTCCATGGTGGTGAAGAC |

**Table S3.** Primary antibodies used in western blot

| **Gene** | **Product number** | **Dilution** | **Manufacturer** |
| --- | --- | --- | --- |
| SV40LT | 15729S | 1:1000 | Cell Signaling Technology |
| **Gene** | **Product number** | **Dilution** | **Manufacturer** |
| h-TERT | ab32020 | 1:1000 | Abcam |
| α-SMA | Ab5694 | 1:1000 | Abcam |
| Collagen-1 | GB11022 | 1:1000 | Servicebio |
| Fibronectin | 26836 | 1:1000 | Cell Signaling Technology |
| GAPDH | Abs830030 | 1:5000 | Absin Bioscience |
| SLC1A5 | 8075S | 1:1000 | Cell Signaling Technology |
| IGF2BP3 | A23295 | 1:1000 | ABclonal |
| Histone H3 | A2348 | 1:1000 | ABclonal |
| c-Myc | PTR2340 | 1:1000 | Immunoway |

**Supplementary materials and methods**

*Isolation and Characterization of PSCs:* Human primary pancreatic stellate cells were isolated from fresh pancreatic cancer tissue and immortalized through transfection with the SV40 large T antigen (SV40 LT) and human telomerase reverse transcriptase (hTERT). The characterization of PSCs was conducted by observing cell morphology, detecting cytoplasmic lipid droplets using the oil red O assay, and confirming positive immunofluorescence staining for α-smooth muscle actin (α-SMA). All studies involving human-derived pancreatic stellate cells were approved by the Medical Ethics Committee of Sun Yat-Sen Memorial Hospital of Sun Yat-Sen University (SYSKY-2023-491-01).

*Extraction and characterization of EVs*: The cells were cultured in complete medium, and when the cell fusion reached 80–90%, the complete medium was discarded, washed three times with PBS, replaced with serum-free medium, and the supernatant was collected after 48 h. The collected supernatant was extracted by differential ultracentrifugation, that is, centrifugation at 500 ×*g* for 15 min and 10,000 ×*g* for 30 min, and then EVs were isolated by ultracentrifugation at 110,000 ×*g* for 70 min. Extracted EVs were dissolved in PBS. Freshly extracted EVs from pancreatic cancer parental cells and GEM-resistant cells were diluted in PBS and then characterized by transmission electron microscopy (TEM). The nanoparticle tracking analysis (NTA) was determined by Nova Biotech (Guangzhou, China). Specifically, for TEM, 5-10 μL of EV samples were applied to a copper grid and incubated for 10 minutes, after which excess liquid was removed. Negative staining was performed by adding 5-10 μL of phosphotungstic acid for 3 minutes, followed by air drying. Imaging was then conducted to evaluate the morphology and size of EVs. For NTA, the sample chamber was rinsed with diluent or pure water, and the sample was subsequently diluted to the appropriate concentration. ZetaView software was used to capture real-time particle images and analyze the particle size. EV marker proteins were detected by western blotting.

*Internalization of EVs*: EVs (10 μg) were mixed with diluted red fluorescent membrane dye PKH26 (PKH26GL, Sigma-Aldrich, USA) and incubated for 5 min at room temperature, protected from light, followed by addition of 2 ml of 0.5% bovine serum albumin (BSA) to quench the dye. The labeled EVs were resuspended in PBS and the unbound dye was removed by ultracentrifugation. Finally, the EVs were resuspended in PBS using 0.22 μM filter to remove bacteria and store it at -80 ℃ away from light. PKH26 labeled EVs were added to PSCs and incubated overnight, after which non-endocytosed EVs were washed away. After fixation, the localization of DAPI, actin, and PKH26 was observed under a laser scanning confocal microscope (LSM710, Zeiss).

*RNA interference and plasmid transfection*: Small interfering RNA (siRNA) targeting IGF2BP3, c-myc, and SLC1A5 is shown in Supplementary Table 3 (ObiO, Shanghai, China). A smart silencer targeting linc-ZNF25-1 was purchased from Ribobio (Guangzhou, China), overexpression plasmids of the corresponding genes were purchased from Hanyi Bio (Guangzhou, China), and lentiviral particles overexpressing linc-ZNF25-1 were purchased from Genepharma (Shanghai, China). Lipofectamine 3000 (Invitrogen, New York, USA) was used for transient transfection according to the manufacturer’s instructions. Stable lentivirus-mediated transfection was performed according to manufacturer's instructions. qRT-PCR or western blot analyses were used to verify the effects of knockdown or overexpression.

*Nucleocytoplasmic separation*: Nucleocytoplasmic separation was performed using NE-PER™ Nuclear and Cytoplasmic Extraction Reagent (78833, Thermo Fisher Scientific, USA). Briefly, the collected cells were incubated on ice in cell separation buffer for 10 min. After centrifugation at 500×*g* for 5 min, the supernatant was collected as the cytoplasmic fraction. The nuclear precipitate was resuspended in cell division buffer and incubated for 30 min at 4 °C. The nuclear fraction was obtained by centrifugation at 12,000 × *g* for 10 min to remove the insoluble membrane debris. qRT-PCR was performed to detect the expression of GAPDH, U6, and lincZNF25-1 in the cytoplasm and nucleus of pancreatic cancer cells.

*RNA immunoprecipitation and pull-down analysis*: RNA immunoprecipitation was performed using an RNA Binding Protein Immunoprecipitation kit (Giese Biotech, Guangzhou, China) according to the manufacturer's instructions. Harvested cells were lysed on ice in IP lysis buffer, and protease and RNase inhibitors were added to the lysate. Subsequently, the cells were centrifuged at 10,000 *g* for 10 min at 4°C and the supernatant was collected. Simultaneously, anti-IGF2BP3 and IgG antibodies were conjugated to the protein A+G magnetic beads. The antibody-conjugated magnetic beads were incubated with the cell lysis supernatant at 4°C overnight. The next day, the isolated RNA-protein complexes were washed, purified RNA was isolated for qRT-PCR, and the loading buffer was boiled with magnetic beads to elute proteins for western blotting.

RNA pull down was performed using the PureBinding® RNA-Protein pull-down kit (Giese Biotech, China) according to the manufacturer's instructions. Cells were lysed on ice for 10 min in capture buffer, which includes protease inhibitors and RNAase inhibitors, followed by centrifugation at 14,000 *g*, 4°C for 10 min, and the supernatant was collected. Meanwhile, streptavidin magnetic beads were incubated with biotin-labeled RNA probe (GenePharma, China) for 1 h, and then cell lysis supernatant was added and reacted at 4°C for 2 h. Subsequently, the magnetic beads were washed and boiled in the loading buffer to remove proteins for western blotting.

*Dual-luciferase reporter gene assay*: To generate a dual luciferase reporter gene plasmid for the CRD region of IGF2BP3-interacting MYC, DNA fragments of wild-type and mutant CRDs were synthesized and cloned into the pmiGLO vector. PSCs were inoculated into a 96 well plate to achieve 70% fusion on the second day and, then, transfected with 100 ng of reporter plasmids containing wild-type or mutant CRD (pmiGLO CRD wt and pmiGLO CRD mut) with Lipofectamine 3000, while co-transfecting or not co-transfecting IGF2BP3 overexpressing plasmids or siRNA. After 24 h, firefly luciferase (Fluc) and Renilla luciferase (Rluc) activities were determined using a dual-luciferase reporter gene assay kit (Yeasen Biotech,China). The relative luciferase activity was calculated as the ratio of Fluc to Rluc.

*Assessment of synergy score*: Pancreatic cancer cells were treated with different concentration gradients of L-ASNase (1ug/ml, 5ug/ml, 10ug/ml, 15ug/ml) and GEM (1nM, 10nM, 100nM, 1000nM) individually or in combination for 72 hours, cell survival and drug inhibition were determined by CCK-8 assay. Synergy scores were then calculated using the SynergyFinder web application (https://synergyfinder.fimm.fi). The synergy score > 10 indicates that the two drugs are synergistic; synergy score -10 to 10 indicates that the effects of the two drugs are additive; and synergy score < -10 indicates that the two drugs are antagonistic.

*Establishing orthotopic transplanted tumor of human pancreatic cancer in mice*: Orthotopic transplantation of human pancreatic cancer cells into mice was performed to evaluate the role of linc-ZNF25-1 in pancreatic cancer and the combined effect of L-ASNase and GEM on chemoresistance. MIA PaCa-2 cells (2 × 10^6^) transfected with a luciferase reporter gene were injected into the pancreas of 6-week-old female BALB/c nude mice. Fluorescent images of tumor-bearing mice were captured using a small-animal *in vivo* fluorescence imaging system (Maestro 3.0.0, USA) prior to treatment. When *in vivo* fluorescence imaging showed that the tumors were visible (approximately 3 weeks after cell implantation), the mice were randomly assigned to the PBS, L-ASNase (MCE, HY-P1923), GEM (MCE, HY-B0003), or L-ASNase + GEM groups. The mice were injected intraperitoneally with L-ASNase (0.2 mg/mouse) , GEM (100 mg/kg), L-ASNase and GEM, or PBS (control) twice a week and underwent weekly *in vivo* small-animal fluorescence imaging to observe tumor progression after reaching the experimental endpoint. All mouse studies were approved by the Animal Care and Use Committee of Sun Yat-Sen University (SYSU-IACUC-2024-000245) and followed the National Institutes of Health Guide for the Care and Use of Laboratory Animals. Experimental animal studies were conducted in accordance with ethical standards for animal research.
